# Scaling Effects on Materials Tribology: From Macro to Micro Scale

**DOI:** 10.3390/ma10050550

**Published:** 2017-05-18

**Authors:** Pantcho Stoyanov, Richard R. Chromik

**Affiliations:** Department of Mining and Materials Engineering, Aluminum Research Centre—REGAL, McGill University, Montreal, QC H3A 0C5, Canada; richard.chromik@mcgill.ca

**Keywords:** microtribology, transfer films, NEMS/MEMS, velocity accommodation mode (VAM), scaling effects

## Abstract

The tribological study of materials inherently involves the interaction of surface asperities at the micro to nanoscopic length scales. This is the case for large scale engineering applications with sliding contacts, where the real area of contact is made up of small contacting asperities that make up only a fraction of the apparent area of contact. This is why researchers have sought to create idealized experiments of single asperity contacts in the field of nanotribology. At the same time, small scale engineering structures known as micro- and nano-electromechanical systems (MEMS and NEMS) have been developed, where the apparent area of contact approaches the length scale of the asperities, meaning the real area of contact for these devices may be only a few asperities. This is essentially the field of microtribology, where the contact size and/or forces involved have pushed the nature of the interaction between two surfaces towards the regime where the scale of the interaction approaches that of the natural length scale of the features on the surface. This paper provides a review of microtribology with the purpose to understand how tribological processes are different at the smaller length scales compared to macrotribology. Studies of the interfacial phenomena at the macroscopic length scales (e.g., using in situ tribometry) will be discussed and correlated with new findings and methodologies at the micro-length scale.

## 1. Introduction

Over the last few decades, the advance in new technology to measure friction, wear, adhesion, and surface topography at the micro- and nano- scale, has led to the establishment of fields such as microtribology and nanotribology [1,2,3,4,5,6,7]. These fields concentrate on the study of two surfaces in relative motion where the contact between them has dimensions on the order of microns down to nanometers. Studies of the phenomena that occur for tribology at these length scales are crucial in order to have a better understanding of a material’s behaviour during single and multi-asperity contacts and to improve the reliability of small scale engineering devices, such as micro- and nano-electromechanical systems (i.e., MEMS and NEMS).

While the driving forces for research on microtribology are primarily the engineering applications of MEMS and NEMS, there are also other motivations, including a desire to understand the underlying mechanisms for friction and wear. Despite the large contact size for a macroscopic tribological system, the initial onset of wear still takes place at discrete sites of contacting asperities with length scales similar to the sizes for the entire area of contact in a microtribological experiment. Thus, studies of microtribology are helpful for understanding interfacial phenomena that are difficult or nearly impossible to observe in a traditional large scale wear test.

The materials of primary interest for microtribology are standard engineering materials, including metals, ceramics and polymers. Often the materials chosen for study are those that are used in MEMS and NEMS devices, such as Si, LIGA Ni, Au, TiNi and DLC coatings. However, materials are sometimes chosen that are only candidates for MEMS (e.g., solid lubricants) or simply materials of fundamental interest for tribology, such as metals typically found in macroscopic tribology applications (e.g., steel). Other materials of interest include biological materials. For example, a gecko’s toe is of great interest in science due to its capability of adhering to nearly all surface topographies. Understanding the adhesive and tribological (i.e., friction and wear) mechanisms of such biological systems is a requirement in the bio-inspired design of tribological systems [8]. To understand and measure properties for biological materials, one must design small scale experiments as those used in microtribology.

Nowadays, nearly all research laboratories in the areas of natural sciences and engineering contain equipment that are capable of examining surface topographies, adhesive properties, or friction forces at the microscopic length scales. Such equipment includes atomic force microscopy (AFM), scanning force microscopy (SFM), and friction force microscopy (FFM). Therefore, there is a large quantity of research being conducted on nano-/microscale surface characterization for various materials. However, there are still many questions that remain unanswered regarding the issues that may arise when decreasing the length scale down to the micro- and nano- level. For example, how are friction processes different at the smaller length scales compared to macroscopic friction? Can friction behaviour of a microscopic system help predict the performance of macroscopic systems? What about third bodies (e.g., transfer films and wear debris) at the microscale? What sliding processes are present at the microscale and how do they impact the friction and wear behaviour? What recent advances in experimental methodology have been used to uncover new understanding of tribology for microscopic length scales?

This review paper will focus on the experimental component of microtribology with the aim of understanding how tribological processes are different at the smaller length scales. Examples of the different methodologies at the microscale and at the macroscale will be provided that can be used to answer the questions above. The subsequent section will focus on the definition of microtribology, including normal forces, contact sizes, as well as the influence of adhesion, roughness and third bodies. Following this section, the various applications of microtribology will be discussed and the different failure modes of microsystems will be identified. The remainder of this review paper will focus mainly on the tribological issue of microdevices. Various methodologies for measuring friction and wear will be initially discussed, starting with AFMs and followed by MEMS tribometers, microtribometer, and nanoindentation instruments. Then, examples of in situ tribometry at the macroscopic length scale will be given followed by explanations of how this technique can provide important insights on understanding the sliding mechanisms at the microscopic length scales. After the in situ examples at the macroscopic length scales, the paper will focus on the different materials that have been used for microtribological studies such as biomaterials, silicon based materials, solid lubricants, etc. This section will also include a comparison between the friction values of the materials measured with different microtribological techniques. Finally, the paper will be concluded with a direct comparison of the micro- and macrotribology.

## 2. What Is Microtribology?

### 2.1. Magnitude of Forces

**Figure 1:**
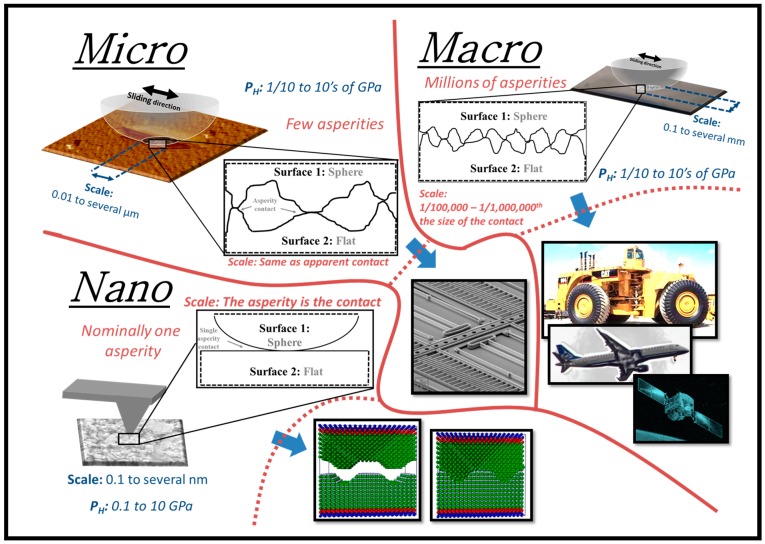
Differences and similarities between nano-, micro- and macroscale tribology in terms of Hertzian contact size/pressure. The contact pressure for all three regimes can be anywhere in the kPa—MPa—GPa range depending on the materials and the loads applied. It should be noted that the term ‘scale’ in the Figure, showing the contact size, represents Hertz contact diameter. In contrast macroscopic sliding contacts, where the contact consists of millions of asperities respectively, in microtribology only few asperities are in contact. At the nanoscale, on the other hand, the single asperity is the contact.

Figure 1 illustrates the overlaps and differences found in literature between macrotribology (conventional), microtribology, and tribology performed using an AFM in terms of the contact pressures and the contact sizes (i.e., Hertz contact radii). Even though some overlaps in contact pressures are observed, the normal forces between the length scales are significantly different; normal forces for nanotribology (i.e., using an AFM) are in the nN’s range, whereas most microtribological studies use forces in the µN and mN range. Higher normal forces have also been reported in literature and referred to as microtribology, but usually such experiments are performed with relatively large counterfaces.

The most common instrumentation, which have been reported to realize normal forces in the µN and mN range consist of scratch-testing instruments, nanoindentation instruments with sliding capability, and commercially available microtribometers. Due to the various instrumentation that have been used for microscopic tribometry, the range for contact pressure in Figure 1 can be further separated into three contact zones in terms of the instrumentation [9]. The highest contact pressures in the microtribometry regime are obtained usually using scratch-testing instrumentation. Such experiments are performed using sharp tips (i.e., R < 1 µm) with the tip moving laterally across the surface and the normal load being linearly increased. The contact pressures of these tests can vary between 7 and 40 GPa. Often scratching tests are performed on coated surfaces to measure the adhesion strength between the coating and the substrate. Sliding experiments using a nanoindentation instrument, on the other hand, are performed with much blunter tips (i.e., tip radius is anywhere between 1 and 500 µm) and therefore lower overall contact pressures (between 0.2 and 7 GPa). In these type of experiments, the tip is either moving laterally in one dimension (i.e., reciprocating wear) or in two dimensions (i.e., scanning wear) with a constant normal force. The lowest contact pressures for microtribological testing are often obtained using commercially available microtribometers with tip radii between 1 and 2 mm, which results in contact pressures between 0.03 and 0.3 GPa.

### 2.2. Scale/Size of Contact

Typical macroscale contacts (i.e., in terms of Hertzian contact radii) that are observed in tribological tests such as pin-on-disk lie in the range between 50 and 800 µm, Figure 1. The approximate range for contact sizes that has been observed in literature and referred to as microtribological experiments is between 0.06 and 6 µm. Most commonly the purpose of such microtribological experiments is to simulate contact conditions that are observed in actual microsystems. However, the range of contacts for microsystems is relatively large (i.e., between 0.003 and 3 µm) and due to limitations within the microtribological instruments, the smaller contact sizes are typically realized using an AFM with a blunt tip (~200 nm). Therefore, the contact area in microtribology can decrease up to five orders of magnitude compared to macroscopic tribology. Such a decrease for contact size and normal load will decrease the real contact from millions of asperities to only a few asperities. Consequently, roughness and actual contact shape will play a larger role [10,11] in the tribological behaviour, which in turn means significant effects on forces such as friction, adhesion, and surface tension.

The effects of the contact size on the friction properties have been previously investigated by McGuiggan et al. [12] using an SFA and an AFM. In this study, two different lubricant films (i.e., perfluoropolyether and polydimethylsiloxane) were examined using contact diameters up to 125 µm. The authors found differences in the friction behaviour between the small and large contact sizes for the same two lubricants; smaller contacts showed similar friction for both lubricants, while larger contacts revealed higher friction for polydimethylsiloxane compared to perfluoropolyether. In addition, the friction value for the larger contact sizes (i.e., SFA measurements using R ~ 1 cm) was an order of magnitude higher compared to the friction of the AFM. Therefore, the results of this study demonstrate a ‘scale effect’ on the sliding behaviour which was influenced by the contact diameter and pressure. More specifically, the authors suggested that the variation in the pressure leads to differences in the local film thicknesses and the mobility of the lubricant resulting in the discrepancy of the friction coefficient.

Similar observations regarding the effect of contact size were also drawn by Yoon et al. [13], who investigated the effects of contact area on the friction of Si and DLC. At the nano-scale (i.e., using an AFM) the authors observed an increase in friction with the normal load and tip size for both kinds of samples (i.e., Si and DLC). A similar behavior of the friction was also observed at the micro-scale (i.e., using a ball-on-flat microtribometer) for the silicon sample. However, the friction of the DLC sample decreased with ball size at the micro-scale. These variations in the friction behavior between the nano- and microtribological set ups were attributed to differences in the sliding mechanisms; while plowing governs the friction at the micro-scale, the friction at the nano-scale is mainly affected by adhesion.

In a more recent paper on micro-scale friction mechanisms, Achanta et al. [14] discussed the influences of contact area on the friction properties. In order to link and correlate data between different length scales, the authors introduced a ‘relative contact size’ parameter α, which is obtained from the ratio of the apparent contact area to the sliding area. Using an AFM, a microtribometer, and a macroscale reciprocating tester, the authors showed that the friction coefficient of dual phase steel against Si_3_N_4_ increased with respect to α for experiments without any wear.

### 2.3. Role of Adhesive Forces

It is well known for design purposes of microdevices that as the size decreases, the surface area-to-volume ratio increases. Therefore, the weight of a miniature device is no more the dominating factor, as observed with large objects [15]. Instead, interfacial forces (e.g., van der Waals forces, capillarity forces, electrostatic forces, etc.) in small devices accommodate the contact and sliding modes. While these forces lead to different failure mechanisms of microdevices, they are also the reason why flies and geckos can move up walls and windows using their many microhairs [8,16].

Generally, for low contact stresses and small contact sizes, adhesive forces have a dominant role on the friction force behaviour. Such adhesive forces arise primarily from van der Waals interactions and/or capillarity effects occurring between the two counterfaces [14]. In a recent paper on nanotribology, Szlufarska et al. [17] reviewed in detail the work of adhesion for single-asperity contacts in terms of the Johnson-Kendall-Roberts (JKR) [18] and Derjaguin-Muller-Toporov (DMT) [19] models. As stated in the review paper [17], both contact modes are valid however, applicable to different contact conditions. The JKR method is accurate for large elastic deformations and short-range adhesive forces. The DMT model, on the other hand, is valid for stiff materials with small contact radii relative to the range of adhesive forces. Even though these models apply to opposite extremes in contact behaviour, both models indicate that the adhesion force acts as an additional contribution to the normal load and therefore, making the total applied load equal to the sum of the adhesive and normal force throughout a sliding experiment [14]. It has also been previously pointed out that the contribution of the adhesive force to the total normal force has a strong dependency on the magnitude of applied normal load [14,20]. Achanta et al. showed that when using normal forces larger than 10^4^ µN, for Si/Si_3_N_4_ tribological systems, the adhesion force becomes insignificant. Similarly, for steel against steel [20], the adhesion contribution was found to affect the friction coefficient at low normal loads but didn’t have a significant influence with higher normal loads. Therefore, both studies confirm the idea that adhesive forces are more dominant on the friction behaviour in microtribology (i.e., small normal loads and contact sizes) compared to macrotribology.

Surface adhesion effects on microtribological properties (i.e., friction and wear) have also been reported for thin solid films [21,22]. For instance, the co-sputtering of metals with MoS_2_ resulted in lower pull-off forces compared to pure MoS_2_, which correlated with improved tribological properties (i.e., lower friction and higher wear resistance). Similarly, the decrease in surface adhesion, due to the addition of MoS_2_ to Au, resulted in higher wear resistance compared to pure Au in dry air. In addition, Barriga et al. [23] studied the influence of humidity on the friction coefficient of Au and Cu coatings. The authors attributed the increase in friction at higher humidity levels (i.e., 50% RH) for Cu-Cu contacts to interfacial energies and capillary forces resisting the movement. More details on this study can be found in Section 5.5.

### 2.4. Role of Roughness–Single vs. Multi-Asperity vs. Continuum

Friction and wear behaviour, at the macroscopic length scale, is normally influenced by the topography of the contacting surfaces. The real contact consists of several micro- and nanoscale contacts to which the overall friction corresponds. For nonelastic sliding contacts (i.e., systems experiencing wear and plastic deformation) the initial surface roughness can have a larger effect on the running behavior rather than on the steady state period. As reviewed by Blau [24], for SiC spheres sliding on Si_3_N_4_ disks, the surface finish did not influence the steady state friction behavior, but surfaces with lower roughness values experienced longer running periods. It is therefore sometimes the case in macrotribology, where the contact consists of millions of asperities, that the contact is treated as a single-asperity contact for simplicity [25,26]. This type of averaging is mainly applicable to specific solid lubricants that follow the Hertzian relationship between the friction force and the normal load (i.e., MoS_2_, DLC). However, for most other contacts in macrotribology, it is essential to calculate the real contact area or understand the single asperity behavior in order to obtain a complete insight on the macroscale tribological properties (i.e., friction and wear) of the system [1].

Experiments with scanning force microscopy (SFM) have been very useful in providing an understanding of single asperity-contacts [17,27], due to their capability of measurements with well defined interfaces. Much of the insights in nanotribology have also been obtained using computational methods such as molecular dynamics (MD). MD is a powerful tool that is used to understand chemical and mechanical changes in single-asperity contacts [17]. Hammerberg et al. [28] demonstrated in an early study how two dimensional MD simulations are capable of facilitating the understanding of interfacial processes such as mechanical mixing and dislocation nucleation. Using Lennard-Jones potentials, Rigney and coworkers monitored structural changes of amorphous and crystalline sliding systems, which they correlated to vortices that were generated by tangential forces [29,30,31,32]. In a more recent paper, Pastewka et al. [33], used realistic potentials to study the chemical/structural changes in diamond contacts. The authors were able to show that the sliding of diamond against diamond results in the formation of an amorphous layer with a growth rate that is dependent on the crystallographic orientation of the first bodies. More of the latest developments and details on similar studies that use single-asperity contacts and MD simulations have been summarized in the review article by Szlufarska et al. [17].

Atomistic simulations are frequently compared to experimental tribology with atomic force microscopy due to the similarities in contact sizes. While such comparisons elucidate interfacial phenomena of single-asperity sliding contacts, it remains a great challenge in understanding how observations at the molecular scale translate onto microscale tribometry. Defining a contact area in microtribological experiments becomes somewhat more complex compared to nano- and macro- tribology. Unlike in nano- and macrotribology, where the contact consists of a single and countless asperities respectively, in microtribology only a few asperities contribute to the contact. Due to the significant reduction in contact size compared to macrotribology, in microtribology roughness plays a larger role and therefore it is crucial consider the real contact area.

### 2.5. Third Bodies

The concept of third bodies, for macroscopic tribology, was initially introduced by Godet in the 1980s [34]. Nowadays this phenomenon is widely accepted for a variety of materials, where the behavior of the third bodies plays a significant role in determining the macrotribological performance (i.e., friction and wear) of the system. For pure metals and metallic alloys it is known that the third bodies typically consist of tribofilms (i.e., mechanically or thermally mixed and deformed material at the surface of each counterface/first-body), transfer films (i.e., similar to tribofilms, but specifically materials transferred from one first body to the other), and debris particles. Previous studies on pure metals have shown that the formation of certain tribofilms decreases friction and wear [35,36]. However, inclusive correlations between the properties of the tribofilm (e.g., thickness, structural, mechanical, and chemical) and the tribological properties have not yet been observed. In terms of transfer films and wear debris, however, the correlations to friction and wear of metals are somewhat more evident. In their book Engineering Tribology, Stachowiak and Batchelor summarised some effects on transfer material and particles on the wear rate and friction behavior of metals [37]. One early understanding of the transfer film behavior in metals (i.e., with mutual solubility) is that transfer particles are collected on the counterface causing the pin to lift up and therefore showing initially a ‘negative’ wear rate [38]. As the pin continues to slide, the transfer material grows larger and is eventually removed causing positive wear. This ends up being a cyclic process that is repeated throughout the sliding process. This idea of the formation and removal of the transfer film was proven to be true from observations of the normal displacements of the tip for the case of zinc against zinc [39].

For macroscopic tribology of solid lubricants, the ‘third body’ process has been investigated thoroughly. For example, in the case of MoS_2_, the third bodies are typically crystalline tribofilms with basal planes oriented parallel to the sliding direction at the worn surface and a MoS_2_ transfer film on the counterface. This phenomenon for MoS_2_ based coatings has been extensively studied at the macroscopic length scales using in situ and ex situ techniques (e.g., TEM, in situ tribometry, Raman spectroscopy) [40,41,42,43,44]. Perhaps the first TEM observation of basal plane orientation of MoS_2_ tribofilms was conducted by Wahl et al. [38] for Pb-Mo-S films. More recently, Scharf et al. [43] investigated the tribo- and transfer film behaviour of MoS_2_/Sb_2_O_3_/Au nanocomposite coatings in dry environment and in air (i.e., 50% relative humidity) using high resolution scanning electron microscopy (HRSEM) and cross-sectional transmission electron microscopy (TEM). The authors observed the presence of crystalline 2H-MoS_2_ basal planes parallel to the sliding direction for the case of dry nitrogen and 50% relative humidity, which indicated a transformation of the MoS_2_ from amorphous to crystalline at the sliding interface. However, Automated eXpert Spectral Image Analysis (AXSIA) maps showed that the overall structure and chemistry of the worn surface was different for the two environmental conditions. For the tests run under dry nitrogen Au nanoparticles were observed to be spread around close to the MoS_2_ basal planes, whereas at 50% RH a continuous ~8 nm thick crystalline Au layer was observed underneath the MoS_2_ tribofilm. The transfer films for both environmental conditions was approximately 1 µm in thickness. In addition, TEM cross-sectional images of the transfer films for both conditions revealed matching MoS_2_ basal planes oriented parallel to the sliding direction, which indicated the ‘basal on basal’ sliding contributed to a low friction and wear.

**Figure 2:**
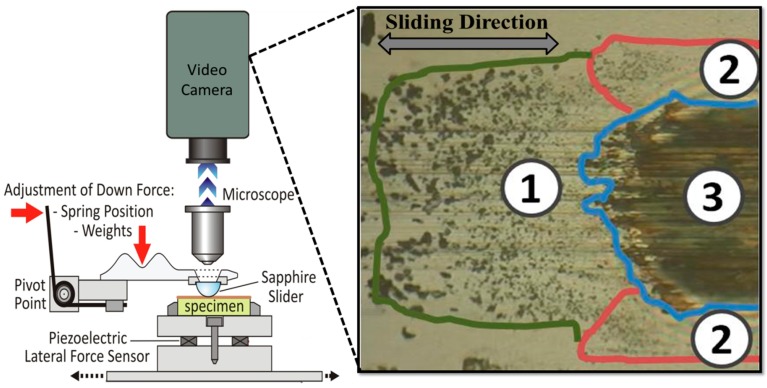
Optical image (**right panel**) showing the contact area of sapphire sphere sliding against Ti-MoS_2_ coating obtained using an in situ tribometer (**left panel**). The image was captured with a microscope monitoring the contact area through the transparent sapphire counterface, as shown on the left side of the Figure. The image shows the three contact zones throughout sliding; (1) the entry zone; (2) the lateral zones; and (3) the internal zone.

The rheology and flows of the third bodies for macroscopic tribology has been described in detail by Descartes et al. [44] and subsequently applied in other tribological systems [45,46]. The authors describe the source flow of the third body (i.e., material trapped in-between the first bodies, not including the ‘structural tribological transformation’ or thermal tribological transformation’ zone) in a natural and artificial form, being formed from an internal and an external source respectively. The internal source includes material detached from the first bodies and the external source consists of artificial material. Throughout the sliding, the material trapped in between the first bodies circulates back and forward creating an external flow of material. The external flow can be further separated into material that is completely emitted from the contact and material that is re-circulated into the contact. These concepts were largely understood through ex situ observation of tribocontact surfaces, but were more explicitly observed and quantified by in situ tribometry (see Figure 2), which will be discussed in more detail in Section 4.5.

Understanding the rheology and flows of the third bodies in a tribological system is important. In general, the contact, including transfer material, can be divided into three zones; the entry zone, the internal zone, and the lateral zones [44], Figure 2. The entry zone is located in front of the contact and the lateral zones are located on both sides of the contact. The entry and lateral zones consist of material (e.g., debris particles) that is formed from the internal/external source and/or re-circulating material. The internal zone, on the other hand, is where most of the contact occurs. In literature, the material in this zone is normally referred to as transfer film.

The third body behavior for microtribology is not understood as well as in macrotribology. As it can be speculated, the rheology and zones of the third body in a certain tribological system are influenced by the contact geometry and applied normal forces. Therefore, when scaling down to microscopic tribology, the flow of the transfer material may be different than macroscale. For instance, such a decrease in contact area can have a significant effect on the external source flow. It is also likely that environmental condition (e.g., external source) have a greater effect on the third body with the decrease contact area. Similarly, since roughness plays a larger role in the tribological behavior (i.e., friction and wear) with the smaller contacts, it is essential to clarify how the internal source flow of the third body changes. These issues are covered in the subsequent sections in more details.

### 2.6. Friction Behaviour at the Microscale

Due to the differences in normal forces and contact areas, the general macroscopic laws of friction are not always applicable for nano-/microscale contacts, as summarized by Mo et al. [47] in Table 1. According to Amontons’ law, initially it was accepted that for most macrotribological systems the friction force is independent of the contact area. However, it was later noted that due to the roughness of the surfaces, the actual area is smaller than the apparent area and consists of a large number of asperity contacts. Therefore, it was shown that the friction force for macro-tribological systems is proportional to the true contact area as shown in Table 1 for Bowden and Tabor’s friction law. With nano-/microtribology, on the other hand, adhesion effects start to play a major role in the friction behaviour, as discussed in Section 2.3, and thus other theories are being considered. The single-asperity theories for nanotribology are shown in Table 1 in terms of the friction force relation to the contact area and the normal load. For nonadhesive contacts, the friction force is proportional to *L*^2/3^ and for adhesive contacts the behavior between the friction force and the normal force is sublinear.

Improving systems with microscale sliding contacts requires a better understand of how the relationship of the friction force to normal force varies for contacts between single asperity and macroscopic length scales as well as identifying additional components that contribute to the the friction force in microtribology. For some instances, non-adhesive laws (e.g., Hertzian model) can be considered for microtribology as long as the adhesive force is less than 5% of the normal load. Otherwise, based on the ‘adhesion map’ of Johnson and Greenwood [48], theories that take adhesion into account may be applied (e.g., Bradley, DMT, Maugis-Dugdale, or JKR).

**Figure 3:**
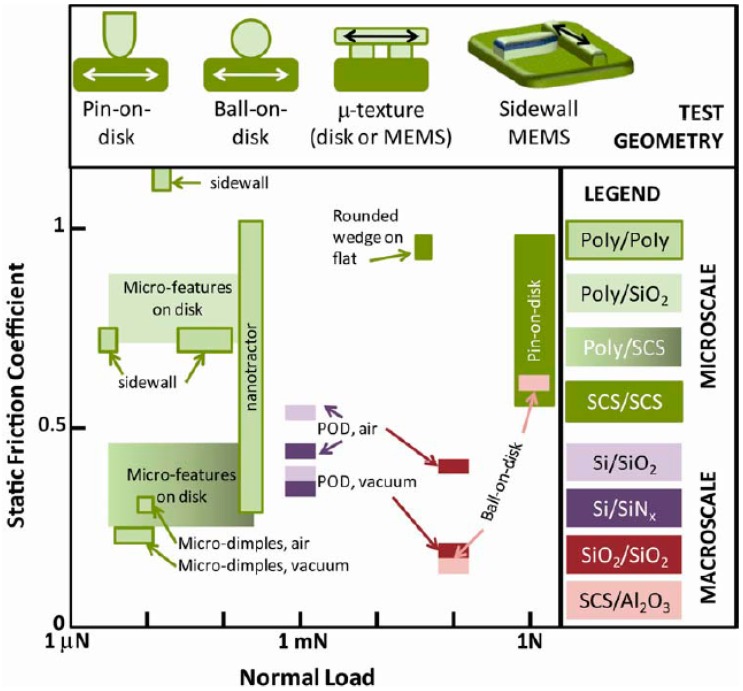
Comparison of static coefficient of friction for micro- and macroscale tribology over a range of normal loads, adapted from reference [9].

Besides the deviation of macroscopic friction laws, when decreasing the contact size down to few micrometers, there are also variations in the friction values. Figure 3 illustrates the differences in the static friction for macrotribology and microtribology over a wide range of normal loads. Various configurations for both length scales are summarized on the upper portion of the Figure and the data ranges are shown on the bottom. The data in this Figure represents the static friction (i.e., the force resisting the initial sliding motion) for the different contact geometries, environmental conditions, and materials. It is observed from this figure that macroscopic friction values are generally lower compared to microscopic values. However, it should be noted for the values in Figure 3 that it is not always possible to determine the exact contact geometry (e.g., contact area, contact pressure) due to the roughness effect and deviations of ideal counterface shapes at the microscale. For example, researchers using MEMS tribometers are much more confident in measuring the static friction values rather than the kinetic friction and have difficulties determining actual contact area in terms of the number of asperities in contact and their size. This is one of the reasons Figure 3 compares macro- and microtribology using the most confident values (i.e., the static friction coefficient and the normal load).

## 3. Application of Microtribology

The interest in studying micro- and nano- tribology became significantly greater over the last few decades due to the advances in the production of microelectromechanical systems (MEMS). Such micro-devices are possible to be produced using silicon photolithographic process as well as a number of other micromachining methods that have recently been developed such as microcutting, microdrilling, micromilling, laser machining [49]. Using such fabrication methods, numerious successful MEMS have been designed, which are currently being used commercially; pressure sensor and air bag sensor for automotive industry, TI digital mirror display [50], RF MEMS capacitive switch [51], Inkjet nozzles (HP), micro-gears, etc. There are also many potential applications for micro devices for applications in automotive, aerospace, and for medical instrumentation [1].

It is generally accepted, from the reliably perspective of MEMS, that these microdevices can be separated into different classes [52,53]. Class I consists of microdevices with no moving parts such as pressure sensors, accelerometers, strain gauge, etc. Class II MEMS are devices that have some moving parts, however, without any rubbing or impacting surfaces (e.g., comb drives, resonators, filters, etc.). Class III devices are more complex in that they consist of moving parts and impacting surfaces (e.g., valves, Texas Instruments Digital Micromirror Display (TI DMD), pumps). The most complex microdevices (Class IV) are those that contain moving parts, impacting surfaces, and rubbing surfaces. Such devices include optical switches, shutters, scanners, locks, etc. This class is the most exciting one for the tribology community since there exist many opportunities for research and development.

Due to today’s available fabrication techniques for the development of microdevices, there are limitations for the material selection of their components. Therefore, the most common materials used in MEMS include polycrystalline silicon, silica (SiO_2_), silicon carbide (SiC), single crystal silicon, sub-stoichiometric silicon (SiN_x_), and alumina (Al_2_O_3_). Based on the material properties of these materials and the operating conditions to which they are exposed (i.e., environmental, contact pressure, contact area, etc.), Merlijn et al. [54] summarized some of the most common failure mechanisms, Table 2. Possibly the most common and unavoidable failure mechanism of microdevices, from this list, is stiction. The main types of stiction consist of solid bridging, capillarity forces, van der Waals forces, and electrostatic forces. Solid bridging is typically formed after the rinsing and etching stage and results in permanent adhesion [55]. While capillarity forces are present mostly at ambient/higher humidity levels or with trapped capillarity liquids, van der Waals forces are present in materials under most conditions.

## 4. Techniques in Micro-/Nanotribology

### 4.1. Atomic Force Microscopy

Atomic force microscopy (AFM), scanning force microscopy (SFM), and friction force microscopy (FFM) have been widely used to study the adhesion, friction, and wear phenomena at the nano-/microscale [1,2,3,17,56,57,58,59]. Such techniques have significantly contributed to our current understanding of fabrication phenomena and failure mechanisms of NEMS/MEMS. Typical Hertzian contact diameters observed with AFM’s, however, range between 0.001 and 0.02 µm whereas contacts in MEMS are observed anywhere between 0.004 and 4 µm [9]. Traditional macroscopic tribometers (e.g., pin on disk), on the other hand, operate in a range with Hertzian contact diameters above 50 µm [9]. Therefore, there is a large gap between AFM based tribometers and macroscopic tribometers for studying the contact behaviour of MEMS. This has led to an increased demand of developing instrumentations that are capable of simulating contacts that fall in the rage of “bridging” the gap between AFMs and macro- tribometers.

An early approach to span the gap between the contacts of atomic force microscopy and macrotribometers was performed by Ducker et al. [60]. In this study, the authors describe the principle of using an atomic force microscope to measure the force between a planar surface and a colloid article. The authors used an AFM with a glass sphere as a tip, having a radius of 3.5 µm, which was attached to the free end of a V-shaped silicon nitride cantilever [60]. A popular development was the ‘nanotribometer’ that was developed with the purpose of exploring the friction and wear behaviour under contact conditions that lie between AFMs and macrotribometers (i.e., contact ranging between 1 nm and about 10 µm) [61]. The design of this tribometer is based on a scanning force microscope developed by the authors. Their instrument overcomes many of the limitations that are observed with standard SFM (e.g., normal load control during a scratch/wear test, thermal drift and creep control of piezoelectric, and a wider range of tip and normal load variations). The authors illustrate the capabilities of this nanotribometer by performing high-cycle wear tests on poly-carbonate, friction measurements on mica, and indentation tests on polyethylene [61].

### 4.2. MEMS Tribometers

Possibly the most effective way to study the friction and wear phenomena of microsystems is to build actual MEMS test platforms that are capable of measuring friction. One advantage of using MEMS as miniature tribometers over AFM based tribometers is that such systems are capable of simulating contact and sliding conditions virtually identical to real microdevices. Another advantage of such MEMS tribometers is that the friction and wear behaviour of such devices can be monitored in situ in a desired environment. Table 3 summarizes some of the MEMS tribometers and their capabilities. For comparison, the ranges in normal loads and static frictions of these microsystems are presented in Figure 3.

One of the first studies on using a MEMS system designed as a miniature tribometer was reported by Lim et al. [62]. This structure was fabricated using a five-mask process. The normal load in this device was generated by an electrostatic attraction between an overhanging structure and an underlying electrode. The friction was measured using the restoring force of a displaced spring [62]. The authors measured the static friction of polysilicon—Si_3_N_4_ and polysilicon—polysilicon interfaces using this MEMS tribometer. The friction coefficient for both interfaces came out higher than expected (i.e., 4.9 ± 1 for polysilicon on polysilicon and 2.5 ± 0.5 for polysilicon on Si_3_N_4_) however, the authors identified some sources of uncertainties and suggested addressing the issues in future measurements. Shortly after this publication, several other MEMS tribometers were designed for microtribological studies of materials used in micro-devices [63,64,65,66,67].

A MEMS tribometer developed by De Boer at al. consists of a surface-micromachined inchworm actuator (i.e., Nanotractor) that provides high-performance characteristics and permits the study of friction and wear at the micro-length scale (second row in Table 3) [68]. In the last few years, researchers have successfully used the Nanotractor design in order to obtain insights on asperity contacts and microtribological behaviour of MEMS materials [68,72,73,74,75,76]. The Nanotractor is capable of operating in a bi-directional motion with a large lateral displacement range (100 µm) at high forces and velocities (up to 0.5 mN and 4.4 mm/s respectively). It consists of two frictional clamps on each side spanned by an actuation plate in the middle. Using a clamping sequence with different phases, back and forward motion is achieved in 50 nm steps. The contact in this device is generated thought the ‘inchworm foot’, which is located in the nanotractor clamp area.

Zabinski and co-workers published several articles on using a MEMS electrostatic lateral output motor for investigating tribological performance of various lubrication strategies at the micro-scale (see Table 3) [69,77,78,79,80,81]. This MEMS tribometer is able to provide a significant amount of information on wear behaviour at the micro length scale due to the numerous contact locations. The slider consists of several dimples, which provide the contact with the counter surface. The cantilever is composed of a gold on polysilicon bimorph and due to the stress in the gold it is therefore naturally curled up [79]. Once voltage is applied to the system, the cantilever curls down and provides a lateral motion to the slider. Wear information after the sliding tests can be obtained from the hinge area and also from the surface underneath the slider. This MEMS electrostatic lateral output motor has been used to study microtribological performance of lubricants such as bound/mobile hydrocarbon-based lubricants, Ionic-liquid lubricants, octadecyltrichlorosilane (OTS) self-assembled monolayer (SAM) coatings, and diamond like carbon (DLC) coatings.

The polysilicon MEMS sidewall tribometer has been extensively used for research on trying to understanding the precise physical process that causes wear of silicon at the microscopic length scale [70,82,83,84,85,86,87,88], Table 3. This device was developed by Senft and Dugger and was the first microdevice that was capable of measuring kinetic friction operating at realistic MEMSs contacts and velocities [52]. The sidewall tribometer was fabricated using the Sandia National Laboratories SUMMIT process. The motion in the orthogonal directions in the sidewall tribometer is created using two electro-static comb-drive actuators. The friction response in this device is calculated using automated image analysis of the displacements of the movable portion [52]. This method for measurements allows the sidewall tribometer to be used for friction responses of MEMSs materials for a high number of cycles. Therefore, it has been extensively used for microtribological studies of monolayer coatings and thin hard coatings at various environmental conditions.

The Leiden MEMS tribometer (see the fifth row in Table 3), which was developed by Van Spengen et al. [71] is similar in design to the trbometers developed by Senft et al. [70] and Tas et al. [85]. It is different compared to the one presented by Senft in the sense that the normal force in the Leiden tribometer is generated by pushing the slide against a counter surface. The friction force in this MEMS tribometer is generated in a similar way to standard friction force microscopes (FFM). The authors also showed how different tip shapes can be used with the Leiden MEMS tribometer. In 2007, van Spengen et al. published some preliminary friction results with varying normal loads using the tip shape called ‘end of a beam’. With low normal loads (i.e., 0.92 µN), the authors observed a stick-slip motion, which was related to the roughness of the counter surface (i.e., sidewall) within the MEMS tribometer. In addition, under these low-load conditions, no wear was observed on the sidewall or the counterface. With higher normal loads (i.e., 1.42 µN), however, some wear was observed and the stick-slip behaviour disappeared. As a future outlook the authors mentioned that with some minor modifications, the Leiden MEMS tribometer has a great potential for investigating more complex nano-tribological effects in micro-/nano- systems.

### 4.3. Microtribometers

#### 4.3.1. Custom-Built Microtribometers

Conducting experiments using MEMS devices that are constructed as miniature tribometers is beneficial in the sense that they provide conditions that a real device would experience. However, MEMS tribometers typically require extensive investment in the fabrication and a great deal of care in order to obtain precise results. Simply for polycrystalline silicon, wide variations in friction values for have been reported with such MEMS microstructures (i.e., static friction values ranged between 1.0 and 4.9 and dynamic friction values ranged between 0.15 and 1) [62,70,89,90]. The differences in friction values between these studies can possibly be attributed to a variation in surface preparation, fabrication methods, contact pressures, environmental condition, and extraction of friction data from the device [69]. In any event, the scatter in the friction results obtained with MEMS tribometers makes it difficult for a MEMS designer to choose the right materials and fabrication method [69].

An additional disadvantage of MEMS tribometers is that any desired variations (e.g., contact size, contact pressure, and materials) within the experiments will require new fabrication procedures and additional devices to be made. Therefore, experiments seeking to explore a range of contact conditions either become costly with MEMS tribometers or require innovative designs. Thus, when exploring a range of experimental variables, one often turns to either commercially or custom-built microtribometers. For example, Gee et al. [91] developed a cost-effective systems for testing of tribological properties at the micro- scale, with the ability of performing in situ tests using scaning electron microscopy. The authors used a lever arm design, which resulted in a tip motion that was an arc of a circle. The angular deviation of the tip, however, was not significant (i.e., 0.03° for a vertical movement of 10 µm) and therefore did not influence the results. Besides being able to use different tip radii with this microtribological system, it also allows for a wide variation of normal loads (i.e., between 12 and 300 mN). In addition, all materials that were used for the components of the tribometer were selected to be compatible for operation in an SEM. The authors published some preliminary microtribological results using this system on hard metals (i.e., WC/C) and some solid lubricants [91].

More recently, in the same laboratory, Gee et al. designed a different microtribometer which is capable of operating on the ‘laboratory bench’ or in a scanning electron microscope [92]. To validate the instrumentation, experiments were carried out on several commercially available coatings. In a later study, the same apparatus was used to simulate abrasion experiments on hardmetals [93].

Liu et al. [94] developed a micro-scratcher in order to study the scratching mechanisms and wear debris generation. The set up of this instrumentation allows for performing various track patterns such as uni-directional, cyclic, square, and intersecting. The micro-scratcher consists of a movable x-y stage and a tip holder on a balanced cantilever arm. The normal force is applied above the tip holder using calibrated weights. In their study, the authors used a silicon cube corner tip to investigate the mechanism of wear-debris generation of polyethylener terephthalate (PET).

Several labs have also developed microtribometers that operate in vacuum chambers [95,96,97,98,99,100,101]. The main motivation of such a set up has been to perform MEMS research. For example, Kosinskiy et al. [95] developed a vacuum microtribometer with the purpose to study the microtribological behavior of Si-Si tribocouples for applications in microsystems operating in vacuum environments, such as the vacuum-based nanopositioning and nanomeasuring machine (IMM). In addition to developing vacuum microtribometers for purely MEMS research, such vacuum-based set ups have also been used for providing a better understanding on the environmental effects on tribological performances [96,102,103] and reproducing/validating atomistic simulations [104].

#### 4.3.2. Low Load Tribometers

A microtriboapparatus was developed by Liu et al. [105] that allows for studying adhesion and friction properties at the micro-scale. The reciprocating motion of this apparatus in the X- and Y- direction is provided by two piezoelectric devices (henceforth “piezo”). Similarly, samples engagement and normal load is accomplished using a piezo in the Z- direction. The advantages of this tribometer over atomic force microscopy are that it can operate at higher velocities (i.e., max velocity can reach 3400 µm/s) with larger tip radii and MEMS components can be directly mounted onto the stage for microtribological measurements. The authors presented the reliability of this microtriboapparatus by studying the surface adhesion and microtribological properties of Si(100), diamond-like carbon (DLC), and hexadecane thiol (HDT). In their study, Liu et al. [105] found that the DLC and the HDT reduced the surface adhesion and the friction force compared to Si(100), with the HDT showing the best performance (i.e., lowest adhesive and friction forces). A comparable microtribometer was also used by Sawyer et al. for studying the friction behaviour of commercially available contact lenses [106], of living endothelial cells [107], and of multi-walled nanotube films [108] using glass pins with radii of 7.78 mm. Similarly, Bonnevie et al. [109] used a custom designed low load microtribometer to study the microtribological response of healthy cartilage under varying sliding speeds and contact areas.

#### 4.3.3. Commercially Available Microtribological Instrumentations

Currently there exists many microtribometers designed by a variety of instrument manufacturers. Such a commercially available microtribometer was recently designed by CETR, Inc. [110], with the capability of performing nanoindentations and micro-scratch tests. The normal loads throughout the scratch tests of this instrument can be varied form 0.1 µN to 0.1 kN. Gitis et al. [110] demonstrated the performance of this microtribometer by evaluating adhesion and friction properties of DLC coatings using a Rockwell diamond indenter with a 200 micron tip radius. Similarly, Ding et al. [111] used a CETR tribometer to evaluate the influence of laser treatment on the microtribological behavior of a-C films.

Another commercially available technique for measuring tribological properties at the microscopic scale is the CSM nanotribometer [112]. This tribometer is composed of three stepper motors (two in the *X*- and *Y*-axis and one in *Z*-axis) [113]. The coefficient of friction is determined by monitoring the deflection force on the cantilever. This nanotribometer is capable of operating in a reciprocating and a rotating mode. Using this instrument, in reciprocating mode, Barriga et al. [23] studied the microtribological properties of sputter deposited gold and copper for potential applications in RF MEMS. More recently, Sahoo et al. [113] used this nanotribometer to study the tribological properties of layered MoS_2_ nanoparticles on steel substrates at the micro-scale. Henry et al. [114] studied the microtribological wear mechanisms of titanium and aluminum nitride coatings using the CSM Instruments Micro-Combi-Tester. Similarly, Zhao et al. [115] used this instrumentation to investigate the microtribological mechanisms of tungsten and aluminum nitride films. Besides applications in metallic and ceramic tribosystems, the CSM microtribometers have also been used for biomediacal applications, such as human tooth wear [116,117].

Basalt-Must (Tetra Ilmenau) is another commonly used microtribometer. It has been mostly used in a sphere-on-flat set up although the tip shape/size are easily modified. The applied normal load as well as the lateral forces are measured from the deflection of a double-leaf cantilever by means of a fiber-optical sensor [118]. More detail on this microtribological instrument can be found elsewhere [119]. This microtribometer as well as modified versions using the same cantilever has been used to study a multy-disciplinary range of topics including contact lenses [120], dental tribology (i.e., tooth wear) [121], graphene [118,122], vanadium carbide coatings [123], W-S-C films [124] and UHV tribometry of silicon [96] silver coatings [102] and tungsten carbide [104].

### 4.4. Nanoindentation Instruments

**Figure 4:**
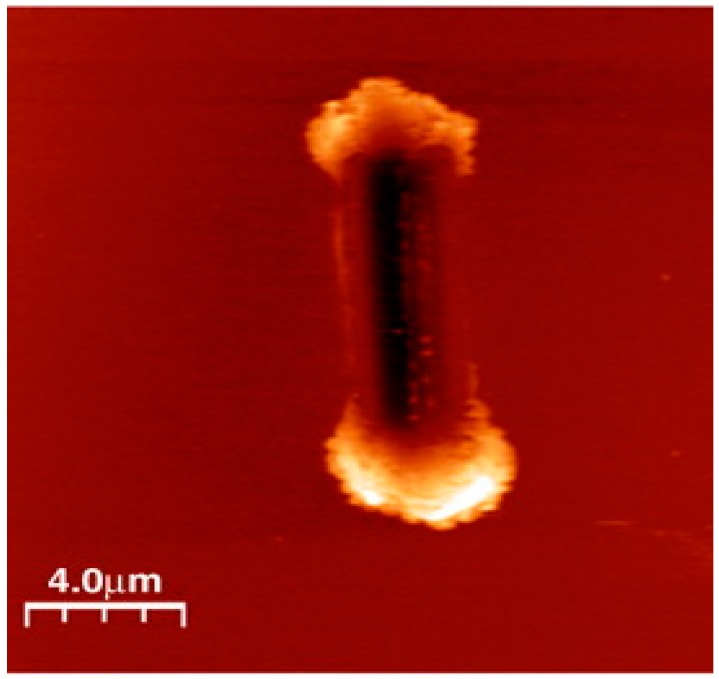
A typical image of the wear track that has been created in reciprocating motion (1D) using the Hysitron nanoindentation instrument. Typically, a spherical diamond or sapphire tip is used in such wear tests with the Hysitron systems.

The Hysitron nanoindentation platform is another widely used instrument that has the capability of measuring the mechanical and tribological properties of materials at the microscopic length scale. This instrument operates with normal and lateral force loading configurations using a patented three-plate force displacement transducer [125]. The force in this system is applied electrostatically, which results in pulling down the center plate towards the bottom plate. The normal force is calculated from the magnitude of the applied voltage. This design of the transducer allows for measurements to be performed using light loads (≤25 µN). Depending on the design/model of the instrument, the maximum normal load that is applied is 30 mN. Usually, for mechanical properties measurements a diamond Berkovich tip is used and for microtribological a diamond spherical tip can be used. A typical image of the wear track that has been created using this instrument is shown Figure 4.

**Figure 5:**
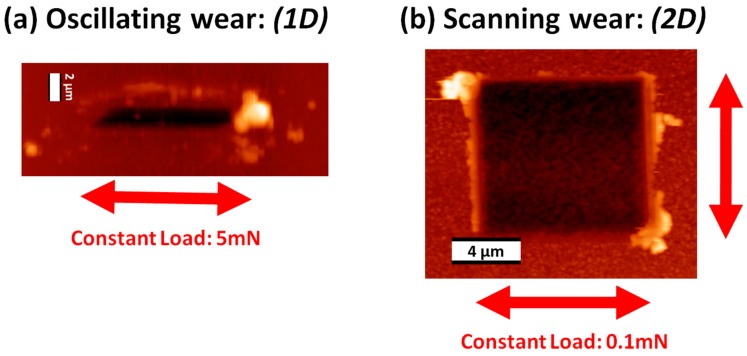
Atomic force microscope images of the (**a**) reciprocating wear test (i.e., 1D) and (**b**) scanning wear test (i.e., 2D).

Similarly, Schiffmann and co-workers [126] explored different techniques for microtribological and nanomechanical testing using a Hysitron nanoindenter. The authors classified the different testing techniques by the dimensions of the lateral movement of the tip; standard nanoindentation with no lateral movement of the tip (0D), scratch and reciprocating wear tests where the lateral movement of the tip is one dimensional (1D), and scanning wear tests where the lateral movement of the tip is in the *X* and *Y* direction with a constant load (2D). AFM images of the 1D reciprocating and 2D scanning wear tests are shown in Figure 5. In their study, the authors performed the comparison between the different testing modes on AF45 glass, single crystal [100] silicon, and polycrystalline aluminium. It was found that the scanning wear tests (i.e., 2D) behaved different from the 1D and 0D tests. This was explained by two main differences in the sliding mechanisms; (1) the exact number of the tip crossing of over a single point on the wear track is much larger and more difficult to be determined in the 2D tests and (2) the piled-up material is redistributed back onto the worn surface, whereas for the 1D test it is re-deposited on both sides of the wear track.

In an earlier study, Schiffmann and Hieke [127] investigated the micro-tribological properties of diamond-like carbon (DLC) coatings using a Hysitron Triboscope. The experimental procedure for the reciprocating wear tests that the authors used consisted of three phases: (1) a pre-scan using a low normal load to measure the height profile; (2) reciprocating sliding at the desired constant load; and (3) a post-scan using a low normal load to measure the resulting wear trace. While varying normal loads between 200 µm and 7 mN, the authors used an equation which contains the elastic (Hertzian) component and an additional plowing term to describe the friction behavior:

(1) $$\mu =\mu_{e}+\mu_{p}=c_{1}L^{-1/3}+c_{2}L^{m},$$

where *c*_1_ and *c*_2_ are constants and m is the plowing exponent. From this equation, it can be observed that the total coefficient of friction is driven by two components; Hetzian elastic contact (*c*_1_*L*^−1/3^) and an additional ploughing component (*c*_2_*L^m^*). The exponent m in the ploughing component is related to the strain hardening index.

**Figure 6:**
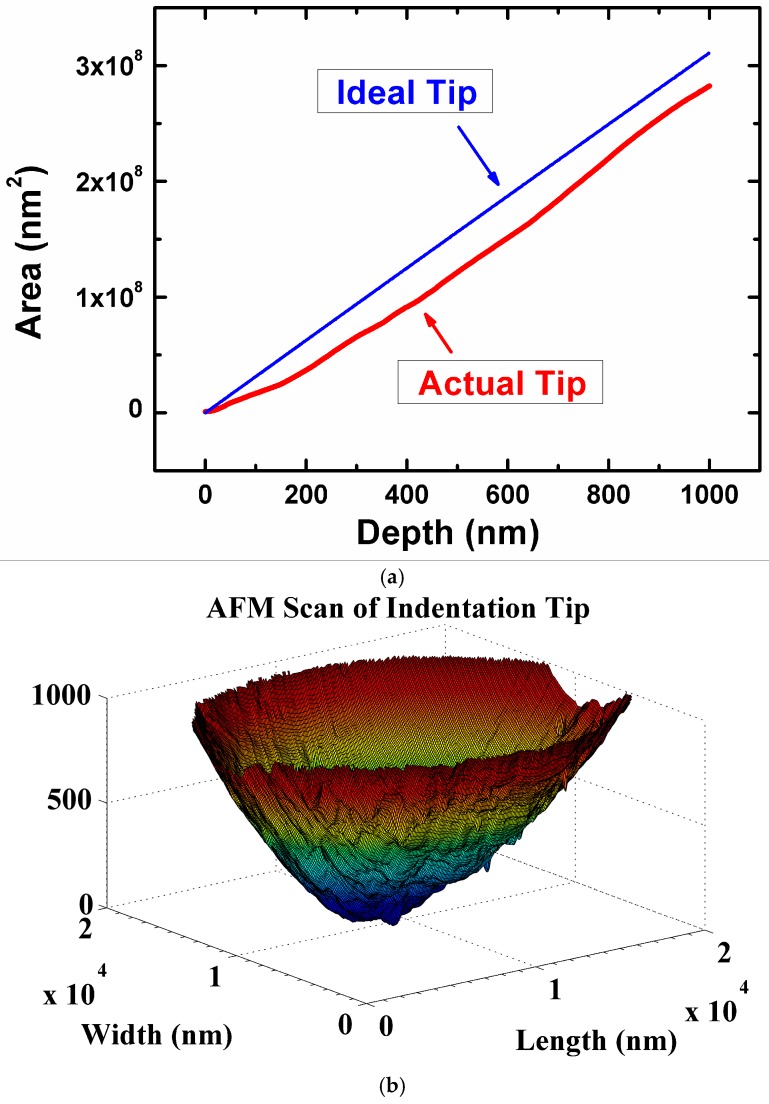
(**a**) Tip area function vs. depth obtained for a spherical diamond tip with a radius of 50 μm; and (**b**) the corresponding tip profile, adapted from reference [21].

The research group led by Chromik used a similar approach to the one of Schiffmann et al. to study the microtribological performances of MoS_2_ solid lubricants for potential applications in microelectromechanical systems [21,22,25,128,129]. The authors measured the friction coefficient using a Hysitron Ubi system with a 2D transducer and spherical diamond tips for varying normal loads. The friction results from the microwear experiments were extracted by the Hysitron software and then analyzed using a custom-build analysis code which was written with Matlab software. For each cycle in these types of sliding tests, the average friction was calculated from the lateral force divided by the normal force and was measured from the central 5 μm of the wear track. The topography and shape of a nanoindentation tip can be characterized using an atomic force microscope operated in tapping mode [21]. Using a custom-built pixel-counting algorithm produced in Matlab, Stoyanov et al. [21] calculated the area function of several nanoindentation tips as a function of depth. The Matlab image of a 50 µm tip is shown in Figure 6.

The topography and shape of a nanoindentation tip is characterized using an atomic force microscope operated in tapping mode in combination with a custom-built pixel-counting algorithm produced in Matlab. This characterization method is useful for calculating the actual contact area and consequently the contact pressure during a given sliding cycle. For certain systems, the contact pressure can then be used to calculate the interfacial shear strength using Equation (2).

When measuring friction for various applied normal loads, it is common to analyze the data using the relationship between coefficient of friction *µ* and the Hertzian contact pressure *P* [42,43,130,131];

(2) $$\mu =S_{o}/P+\alpha$$

where *S*_o_ is the interfacial shear strength and *α* is the limiting coefficient of friction. However, due to deviation in the Hertzian contact behaviour at the microscale [21,22], the pressure *P* is calculated using the real contact area of the tip. The contact area can be obtained from the area function of the tip (i.e., obtained from the AFM characterization) and the elastic depth at the end of the sliding test.

**Figure 7:**
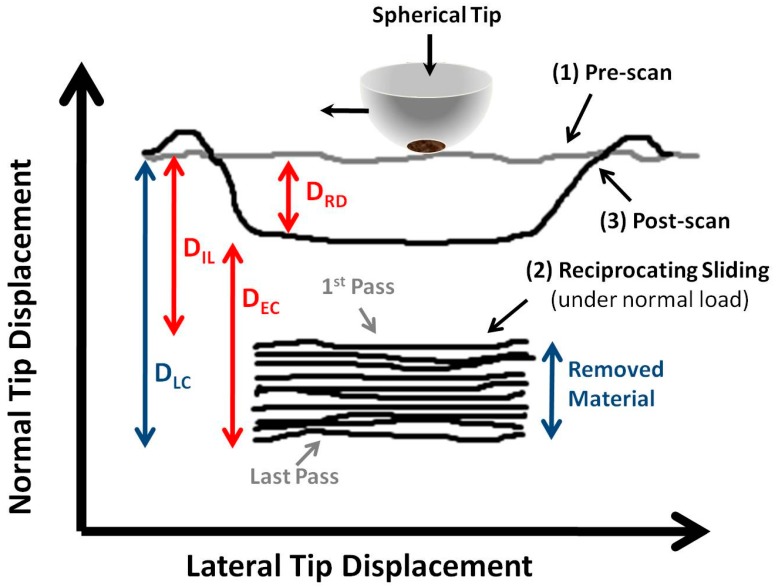
Schematic representation of wear data obtained from a microtribology reciprocating wear test conducted with a Hysitron nanoindentation system, adapted from reference [132].

**Figure 8:**
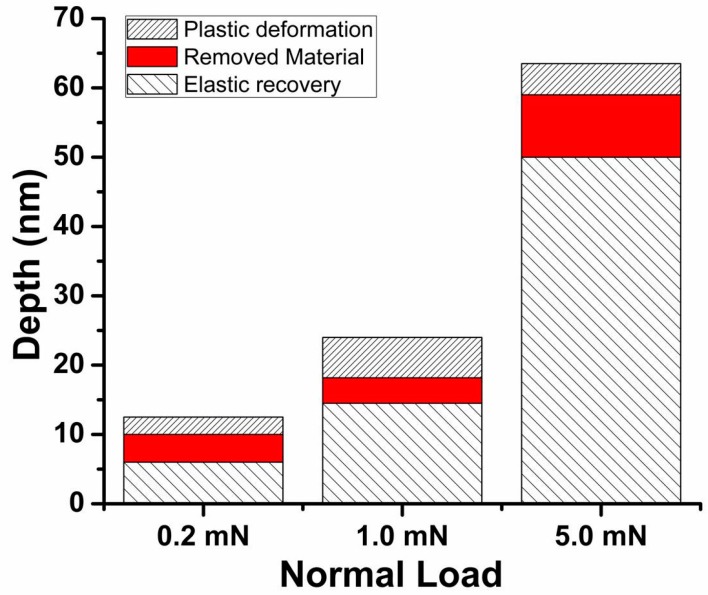
An example of the depth contributions measured from a microtribology reciprocating wear test on a Ti-MoS_2_ coating. The depth contributions are determined from the depths labeled in Figure 6 in the manner described in References [21,132].

Besides obtaining friction with the Hysitron nanoindenter, the wear behaviour of surfaces can also be evaluated by using the three phases during the microtribological tests. A schematic representation of a typical wear test result is shown in Figure 7 in order to illustrate the three phases during the sliding process [132]. In this Figure, D_IL_ represents the initial loading depth, which can be calculated from the elastic-plastic depth of an indentation test or from the post analysis of the test [133]. D_RD_ is the residual depth and is measured from the height difference between the last cycle and the last cycle under a certain load and the post scan. Lastly, D_EC_ is the elastic recovery and is measured from the height difference of the post scan and the last cycle. Using these measurements and the equations presented in reference [22], one can calculate the different depth contributions. An example of the depth contributions is shown in Figure 8 for cosputtered Ti-MoS_2_ coatings.

### 4.5. In Situ Tribometry

The process between the two surfaces in contact (i.e., interfacial process) plays a key role in controlling the friction and wear behaviour of the tribological system. Most studies have used ex situ techniques of separated contacts to investigate the interfacial process. This type of analysis approach might be considered a “CSI” (Crime Scene Investigator), where one examines the worn surfaces after the sliding process and tries to understand what happened. Similarly to a crime scene, however, many of the important aspects in the interfacial process, controlling the tribological properties, have a dynamic behaviour (e.g., plowing, material deformation, chemical interactions, etc.) that cannot be observed by only ex situ techniques. Therefore, tribologists have often developed different surface analytical tools to examine the buried interfaces throughout the sliding procedure. Over the years, such techniques have included optical microscopy, videography, interferometry and chemical spectroscopy (Raman, FTIR) [9].

As recently reviewed by Chromik et al. [134], the first studies on visual observations of the sliding interfaces were performed by Gohor et al. [135] and Foord et al. [136] for lubricated sliding conditions, as well as Sliney for solid lubrication [137]. Following these early pioneering studies, other groups began conducting in situ tribometry, including the groups of Berthier at INSA de Lyon in France [44], the group at Perdue University [138] and the group led by Singer and Wahl at the U.S. Naval Research Laboratory (NRL), where the tribometer developed by the latter had the combined capabilities for visual observations and Raman spectroscopy [42,139,140,141,142]. The observations and Raman microscopy in this tribometer are performed through transparent counterfaces which are typically made out of sapphire or glass. Even though this technique uses macroscopic contacts and while the interfacial phenomena in macrotribology may not directly translate to a microscale sliding contact, it does provide valuable insight into third body processes that likely still occur for microtribology. The objective of this in situ instrumentation was to identify what third body processes and velocity accommodations modes control the friction and wear behaviour of solid lubricants such as molybdenum disulphide, diamond-like carbon, and other nanocomposite coatings.

In situ tribometry has been effective at demonstrating the importance of third bodies in controlling friction and wear, and in general, enforcing the idea that the wear process can be thought of as a tribological circuit [143]. When third body material is retained in the sliding interface, it is often a positive influence on wear resistance, leading to like materials sliding against one another resulting in a potentially low friction interface. This is especially true for molybdenum disulfide—based solid lubricants [25,42,144,145] and some diamond-like carbon coatings [141,146,147], but has even been found to be important for metals and metal matrix composites [148].

**Figure 9:**
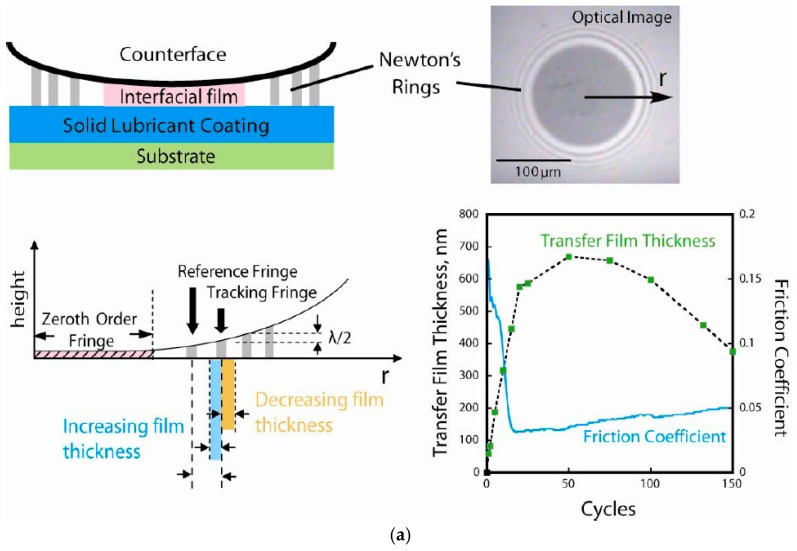

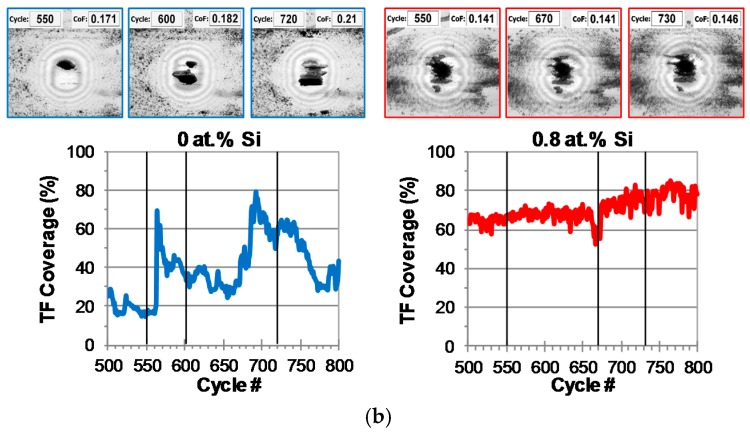
(**a**) Examples of in situ tribometry using a transparent counterface (e.g., Sapphire). The interference fringes are located around the contact region and can be used to quantify transfer film thickness, as shown in the bottom left of the figure. An example of the transfer film thickness measurements using this Newton’s ring method is shown in the lower right panel; Figure 9 (**b**) An example of using image processing of in situ images to explore the retention of third bodies in the contact region for a Ti-C coating (noted as 0 at% Si) and a Ti-Si-C coating (with 0.8 at% Si). In this study it was found that the addition of Si stabilized the transfer film compared to pure a Ti-C coating. (Adapted from reference [134,150]).

In situ tribometry can be used in many ways to analyze third body formation, flows and retention at the sliding interface of a tribocontact. One such method, is to use the Newton’s ring patter that appear in an in situ image of the contact to monitor variations in the average thickness of the transfer film [144] (see Figure 9). On the upper left part of this figure is a schematic representation of the cross-section of a transfer film formed between a solid lubricant and the counterface. The top right of this figure shows an actual view of the contact through a sapphire hemisphere (the slider material) using an optical microscope. This image was taken before the sliding commenced and the dark circle in the center of this image is the contact area, which agrees well with calculations from the Hertzian contact model [149]. The interference fringes are around the contact region and can be used to measure an average transfer film thickness. An example of the transfer film thickness measurements using this Newton’s ring method is shown in Figure 9a. It is observed that the changes in transfer film thickness with respect to the cycle number correlate with the evolution of the coefficient of friction for a Ti-MoS_2_ coating [133], as seen in the lower right graph.

Another method to explore stability of thirdy bodies with in situ tribometry is by conducting an image analysis of the coverage of transfer film material during a sliding test. Figure 9b shows an example for Ti-C and Ti-Si-C coatings, where the transfer film coverage was measured by image processing routines written in ImageJ software. The findings in this study were that the retention of transfer film was enhanced when Si was added to Ti-C and this also was reflected level and stability of the coefficient of friction [150].

Using in situ tribometry, the group found correlations between transfer film characterization and tribological properties (i.e., friction and wear) for various solid lubricants at different sliding conditions. Dvorak et al. [42] investigated the third body process and velocity accommodation modes of Pb-Mo-S coatings using in situ tribometry. The authors found that when sliding on Pb-Mo-S coating, a MoS_2_ transfer film is formed during the first few cycles of the test which contributes to low friction coefficients in dry and humid environment. Furthermore, in situ monitoring revealed that the dominant velocity accommodation mode was interfacial sliding for both conditions. In humid air, however, a second velocity accommodation mode was observed (i.e., debris shearing).

In addition to exploring the dynamic nature of transfer film thickness and coverage, in situ tribometry is also useful for determining velocity accommodation modes [131]. Dvorak et al. studied a Pb-Mo-S coatings using in situ tribometry and found the primary velocity accommodation mode to be interfacial sliding between the transfer film and wear track with some interfilm shearing occurring at higher humidity [42]. Chromik, et al. [145] studied a set of nanocomposite coatings comprised of diamond-like carbon, MoS_2_, Au and yttria-stabilised zirconia (YSZ). With in situ tribometry, they identified the primary velocity accommodation modes to be similar to the study of Dvorak (i.e., interfacial sliding primarily with some interfilm shearing at elevated humidity). However, for coatings with higher YSZ content coatings, a third velocity accommodation mode appeared—abrasive plowing that led to instability in the transfer film and friction spiking [145].

While in situ methodologies within the contact are ideal for understanding third body behavior, they confine the material selection of the counterface to a transparent one [151], which makes it sometimes questionable to extrapolate sapphire vs. material tribology to studies of tribological pairs using “engineering” materials such as the more popular steel counterface. Generally, the differences in adhesion of transfer film material to sapphire versus the adhesion to steel are what determine the degree to which one can quantify third body processes by in situ tribometry for engineering applications. Another limitation for the in situ tribometry technique is for study of opaque materials, like metals. While valuable information can still be gained about transfer films stability and third body flows for metals [148], the sliding interface is not directly observed and most information on the dynamic processes occurring for metallic wear cannot be observed, even with in situ tribometry. Nevertheless, third body processes do occur in much the same way for any tribological contact and insights from the in situ tribometry methods are a useful starting point for understanding their behavior.

**Figure 10:**
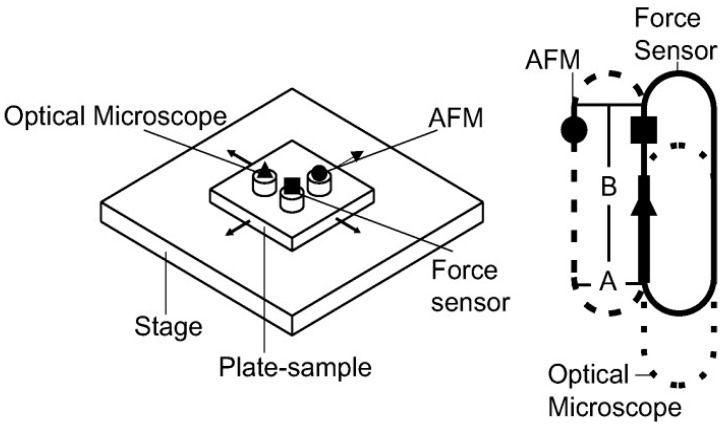
The concept of this instrument which consists of an optical microscope (triangle), pin (square), and an atomic force microscopy (AFM) (circle). On the right, the travel path for the three components is indicated as AFM (dashed line), optical microscope (dotted line) and pin (solid line). [152].

A complementary in situ methodfor the study of metallic friction and wear was developed by a group led by Martin Dienwiebel. The instrument includes atomic force microscopy and holographic microscopy to monitor topographical changes of the worn surface of sliding systems. The most significant advantage of this tribometer is the capability to perform surface topography at the micro- and nanoscale with online measurement of wear and with in situ lateral forces detection [152]. Figure 10 shows the concept of this instrument, which consists of an optical microscope, pin, and an AFM. The image to the right in Figure 10 shows the path for the AFM, optical microscope, and the force sensor and pin. This motion path is designed in this way for the purpose to allow all devices to have access on a specific part of the path (i.e., on the thick continuous line). The pin is attached to the force sensor and is capable of performing smooth cycles without losing contact with the counter surface. The other two sensors (i.e., the optical microscope and the AFM) examine the wear track at the same position each time the pin slides on the ‘thick line’ path shown in Figure 10. Thus, surface topographical images can be obtained after every cycle with a very short pause of the motion. In their first study, the authors performed a preliminary study using this instrument on pure iron samples with polyslphs olephim (PAO) as a lubricant. This study showed promising results in terms of the wear and friction behaviour and the authors concluded that this tribometer can also be used for more complex systems.

**Figure 11:**
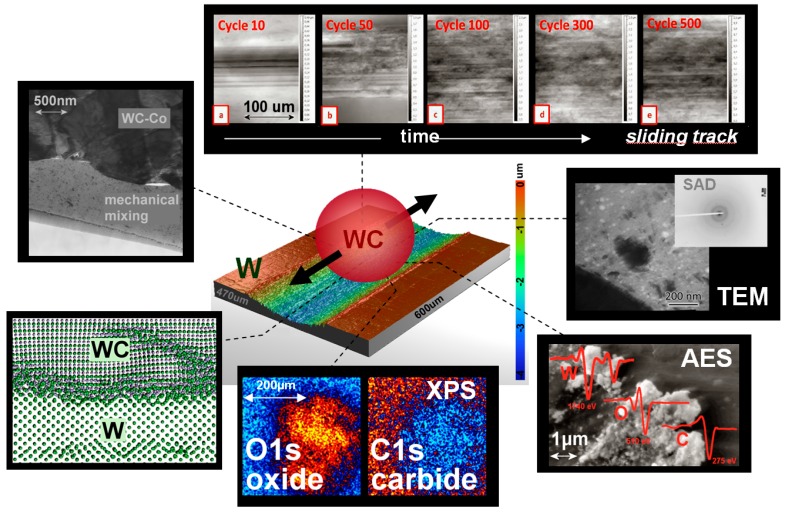
An approach of studying dynamic interfacial processes leading to the variations in the friction and wear. This concept involves linking on line experiments to atomistic simulations with realistic bond order potentials. The structural/chemical changes observed in the simulations are compared to ex situ analysis using transmission electron microscopy, X-ray photoelectron spectroscopy, Auger Electron Spectroscopy, and Raman spectroscopy (adapted form reference [153]).

In order to also capture the dynamic process within the buried interfaces in metallic sliding contacts, in a set of resent studies [104,153], the experimental results of the on line tribometer were linked to atomistic simulations using realistic bond order potentials. Subsequently, the structural/chemical changes observed in the simulations were compared to ex situ analysis using transmission electron microscopy, X-ray photoelectron spectroscopy, Auger Electron Spectroscopy, and Raman spectroscopy. This approach for studying the interfacial process is captured in Figure 11. It is reasonable to argue that such a comparison between atomistic simulations and macroscopic experiments is not possible due to the differences in the environmental conditions. Therefore, to validate this comparison, the MD simulations are reproduced experimentally in UHV. In the first study [104] on WC/W tribocouples, it was observed that the increase in the friction coefficient is attributed to mechanical mixing and amorphization within the WC. Using the same approach in the second study [153], the topographical changes of dry and lubricated sliding contacts were evaluated to identify the different velocity accommodation modes which lead to fluctuations in the friction and wear.

**Figure 12:**
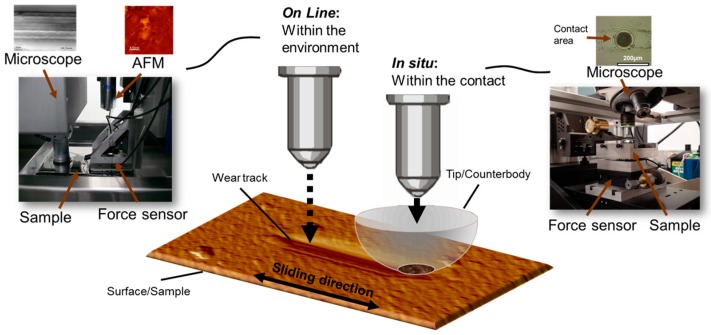
Schematic representation of combining in situ and on-line methods for studying the interfacial processes in sliding couples [154].

In a more recent study, the two approaches (i.e., in situ and online technique) were combined in order to study the interfacial processes in lubricated metallic (i.e., aluminum based) sliding conditions, as shown in Figure 12 [154]. This unconventional approach showed that the evolution of the roughness followed the coefficient of friction trend closely, with initially low values followed by higher roughness during steady state. Similarly, the transfer film behavior correlated well with the roughness of the worn surfaces and the subsurface microstructure of the worn surfaces, as revealed by the in situ technique.

In situ and on line tribometry are powerful tools for understanding interfacial phenomena at the macroscopic length scale, but the working principles of these instruments are difficult and, in some cases, impossible to implement at the microscopic length scale. One of the few examples of in situ methods applied to microtribology was accomplished by Krick et al. [155,156], at the University of Florida. They developed an in situ micro-tribometer that allows for optical observations of the contact size, geometry and topography. In order to validate their instrumentation, the group performed a study on a rough rubber sphere sliding against a glass counterface with varying normal loads between 1 and 50 mN. The results of the loading-unloading experiments revealed a strong hysteresis of the measured real contact area plotted against the normal load. When sliding however, a distortion was observed in the contact geometry as well as an increase in contact area during steady state. In addition to the experiments, the authors used Persson contact hardness mechanics theory [157] to calculate the changes in contact area with respect to the magnification. A limitation of this technique is that information on third body flows and transfer film behavior is still unable to be observed. It has been designed with the primary goal of determining the real contact area.

While it is generally understood that many of the velocity accommodation modes and third body flows presented above for macrotribology also occur for microtribology, all studies of these processes for microtribology have been primarily with traditional post analysis, where the contact is separated and analyzed after the test is complete. Therefore, there still remains an incomplete understanding of the differences in velocity accommodation modes for solid lubricants, metals and coatings mentioned above when decreasing the contact area to a few micrometers. For instance, the transfer film at the microscale is likely to differ in its dimensions; roughness of the contacting surface may play a more prominent role in determining transfer film stability and environmental effects may differ from macroscopic tribology.

**Figure 13:**
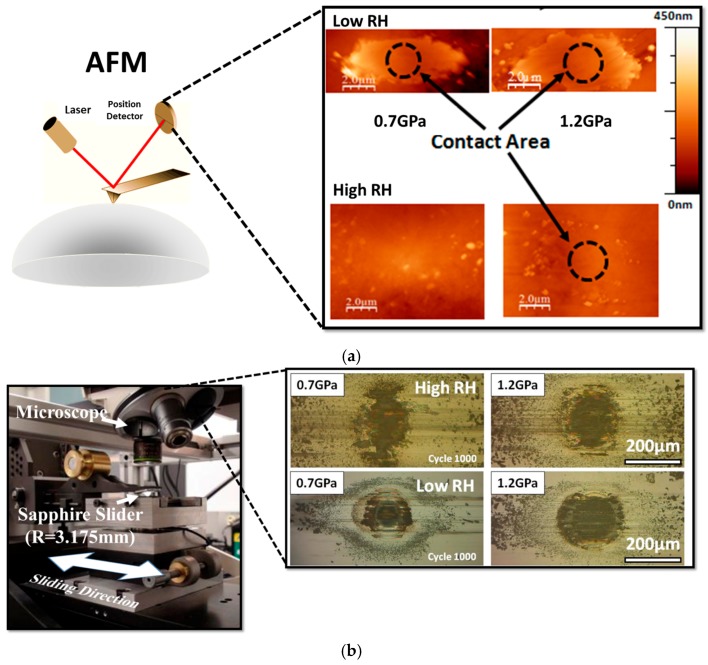
Transfer film analysis for macro- and micro- tribology using in situ and ex situ techniques. For both length scales, the experiments were performed at low (~4% RH) and high (~35% RH) humidity levels. Figure 13 (**a**) shows AFM analysis of the transfer films for the microscale experiments and Figure 13 (**b**) shows the in situ analysis for the macroscale experiments. Both length scale under dry conditions formed transfer films which contribute to a decrease in friction and wear and the main velocity accommodation mode was interfacial sliding (Adapted from reference [25]).

One such example of a study of microtribology with separated contacts was conducted by the tribology group at McGill University. The interfacial process were studied for solid lubricants due to interest for their potential applications in microdevices. In this study, Stoyanov et al. performed a direct comparison between macro- and micro- scale tribology, where for macro- in situ methods were used and for micro- separated contact were examined ex situ [25]. The microtribological test in this study were performed using a Hysitron nanoindenter, whereas the macrotribological experiments were performed using an in situ tribometer. A ‘real time’ study of the transfer film behavior and velocity accommodation modes was possible to be conducted with the in situ tribometer at the macro-scale. At the micro-scale, on the other hand, the transfer films were analyzed ex situ on the counterface (i.e., nanoindentation tips) by means of atomic force microscopy. An example of the transfer film analysis for the two length scales at low (~4% RH) and high (~35% RH) humidity levels is shown in Figure 13. Both length scale under dry conditions formed transfer films which contribute to a decrease in friction and wear and the main velocity accommodation mode was interfacial sliding [25]. However, the presence of humidity led to different velocity accommodation modes for both length scales; transfer film shearing and transfer film removal for the macro- and micro- scale respectively.

## 5. Materials in Microtribology

### 5.1. Biomaterials

#### 5.1.1. Technology Relevance

Exploring the origin of friction and wear properties at the atomic level and the development in the fabrication technology of microsystems were initially the two major drives for studying tribological properties at the small length scale. More recently however, the advance of nano-/microtribometers has raised a great scientific interest in studying the adhesion, friction, and wear properties of biological systems at the nano-/micro-length scales. Understanding these miniaturized properties of biological materials is useful in solving most challenges in further development of complex microdevices [119] as well as a requirement in the bio-inspired design of tribological systems. In addition, for certain biological applications, the microtribological approach tends to be a more simple and robust tool compared to conventional macroscopic set ups. For example, Scherge et al. [121] suggested the use of microtribometry in dental applications and showed how microtribometry can simply help for development and characterization of toothpaste. Recent studies have further verified the convenience of microtribology in dental application by investigating human tooth enamel and artificial hydroxyapatite [116,117]. A similar approach was also adopted by Erickson et al. [158] for dinosaur’s teeth. A more detailed review on micro- and nano- biotribology can be found in reference [119].

#### 5.1.2. Friction and Wear Behavior

Geckos are probably one of the most interesting creatures from the adhesion point of view of biological systems. Their ability to climb up smooth vertical surfaces is due to a combination of adhesive forces and the increased surface-to-volume ratios. From the microscopic aspect, it is the hairy attachment system on a gecko’s foot which makes it possible for geckos to adhere to surfaces [159,160]. A gecko’s foot consists of nearly five hundred thousand keratinous hairs or setae, which are approximately one-tenth the diameter of a human hair [16]. Autumn et al. [16] reported the first direct measurements of single setal (i.e., gecko foot-hair) force using a MEMS force sensor. The authors mentioned that the values of their adhesive force measurements indicate that the individual seta operate by van der Waals forces. It was concluded in this study that the natural mechanism of a gecko’s foot is of great biological inspiration for the development of highly adhesive devices.

A similar conclusion was also provided by a follow up study on nanomechanical measurements of a gecko’s foot adhesion [8]. The authors in this study focused on measuring the influence of atmospheric condition and surface chemistry on the adhesion of a gecko. The results in this study clearly showed that the adhesive forces of a single gecko spatula are strongly affected by humidity on a nanoscopic level. Therefore, the authors suggested that when using this biological mechanism for the design of artificial attachment systems, one should take into consideration the influence of the atmospheric conditions.

Besides the great scientific interest in researching the adhesive and tribological properties of geckos and flies, there have also been a large number of studies on other biological surfaces at the microscopic level. Microtribometers have been shown to be a very useful tool for measuring friction properties of contact lenses due to their challenging contact and sliding conditions [106]. For instance, the research group led by Sawyer at the University of Florida, studied the friction coefficient of soft contact lenses using a microtibometer with dual glass flexures to apply and measure mN-range forces [106]. In this study the authors obtained a friction coefficient in the range between 0.025 and 0.075. The results indicated that the main contributions to the friction forces include viscoelastic dissipation, interfacial shear, and viscous shearing. The authors also developed a model that takes these three contributions into account and was consistent with the experimental data. It was suggested that the main contributions to the friction force was due to viscoelastic dissipation of the contact lens material and interfacial shearing.

#### 5.1.3. Understanding of the Tribology through 3rd Bodies

Sawyer led a different study on tribology properties of biological materials using the same microtribological apparatus as in the study above [107]. The purpose of this research was to measure friction of a single layer of arterial endothelial cells and to obtain a better understanding of the biological response to the tribological stresses (e.g., cell damage). Using a normal force of 0.4 mN and a sliding speed of 300 µm/s, the authors observed friction coefficient values ranging from 0.03 to 0.06. It was also observed that with the lower friction values nearly no cells were removed, whereas with the higher limit of the coefficient of friction many cells were detached. Further investigations with the same dual flexure-based microtribometer were also performed by Dunn et al. [161] using compliant hydrogel counterfaces against live human corneal epithelial cells.

### 5.2. Silicon Based Materials

#### 5.2.1. Technology Relevance

The interest of studying tribological properties of Si and SiO_2_ surfaces at the microscopic length scales is quite different compared to biomaterials. Due to the currently available fabrication technology of MEMS/NEMS, Si and SiO_2_ are the most commonly used materials in microdevices. Therefore, a large amount of studies at the microscopic length scales have been carried out on silicon surfaces using commercially available ‘low load’ tribometers, custom-build microtribometers [103], and MEMS tribometers [162,163,164].

#### 5.2.2. Friction and Wear Behavior

Alsem et al. [88] used an on-chip polysilicon side-wall MEMS tribometer to study and understand the active wear mechanisms under ambient conditions. The authors used scanning and transmission electron microscopy to examine the worn surfaces and local temperature changes were monitored using advanced infrared microscopy. Debris particles were observed, which were created by adhesion and fracture through the silicon grains. Initially, the dominant wear mechanism was adhesion, but subsequently to the formation of the debris particles, plowing tracks were observed on the worn surfaces indicating a second wear mechanism.

Ku et al. [162] studied the wear behaviour of Silicon surfaces using a silicon disk specimen. The disk specimens were 2 mm in diameter and were fabricated using a Deep Reactive Ion Etching (DRIE) technique. Using these devices, the authors showed that friction and wear behaviour of silicon at the microscopic length scale have a strong dependence on the surface condition prior to testing. For example, the highest wear coefficients were seen with surfaces that were cleaned with oxygen plasma prior to testing. On the other hand, surfaces that were exposed to air before testing showed negligible wear and relatively low friction values. Such observations in this study indicated that some element present in air or in water attach to the surface of the silicon during air exposure and act as lubricants (i.e., lubricious contaminant).

Previous studies have also shown that the surface condition of silicon, prior to testing, can have a significant effect on the microtribological properties. Ahmed et al. [165] studied the microtribological properties of silicon, oxides, and carbide surfaces using a ‘precision microtribometer’. In terms of roughness effects, the authors found that if at least one of the surfaces is relatively flat, the friction behaviour is strongly influenced by capillary forces. With the rougher surfaces, on the other hand, the roughness effect showed to decrease the friction. Therefore, it was concluded that the surface conditions (e.g., roughness) for materials used in MEMS need to be studied extensively in order to improve the reliability of microdevices.

#### 5.2.3. Understanding of the Tribology through 3rd Bodies

Sliding on silicon typically leads to the formation of damaging debris particles, which are formed mainly due to breakage of asperities or fractures in the grains. Using SEM imaging of the worn surface, I.S.Y. Ku et al. [166] showed that the debris particle agglomerate and form clusters that are 3–20 µm in size. The authors suggested, based on previous literature [167], that the sliding causes the silicon surface to oxidize, which when removed leads to the formation of amorphous silicon oxide debris particles. The volume of these debris particles have also shown to increase with a decrease in relative humidity [167]. The evidence of grooves on the worn surface in the study of Ku et al. indicated that the presence of such debris particles in the sliding interfaces cause abrasive or plowing wear.

Liu et al. [105] suggested that the tribochemical reaction between the silicon surfaces has also a significant effect on the microtribological properties. High sliding speed leads to a reaction between the silicon oxide on the Si(100) surface with water molecules and therefore results in the formation of a Si(OH)_4_ layer. This Si(OH)_4_ tribofilm layer is known to have low shear strength and thus decreases the friction force.

More recently, a detailed study on the wear mechanisms of polycrystalline silicon was performed using the sidewall tribometer [168]. During the running in period, the authors suggested that the wear process was dominated by adhesive forces leading to a removal of the monolayer coating and the native silicon oxide layer. Subsequently, adhesive wear occurs on the freshly exposed silicon surface, creating debris particles (by fracture in the grains) and changing the wear mechanism to abrasive wear, as also suggested by Ku et al. [162]. Once the steady state stage is reached, the agglomerates are broken down in to smaller pieces and consequently decreasing the surface roughness.

#### 5.2.4. Solutions/Improvements

Debris particles are a common problem in microdevices and can lead to premature failure of the system. One solution to this is to minimize the contacts in a microdevice and adjust the contact pressures and/or sliding velocity to obtain the least amount of debris particles. However, this causes many constraints in the design and therefore is not very efficient. A more effective solution would be to use coatings (e.g., DLC, MoS_2_) that reduce the adhesion and friction.

### 5.3. Diamond-Like Carbon Coatings

#### 5.3.1. Technology Relevance

One of the most commonly tested coatings at the micro scale is diamondlike carbon (DLC) [79,105,110,133,166,169,170,171,172,173,174,175,176,177,178]. The main drive for examining DLC coatings at the micro-scale is due to its desirable properties (e.g., chemical inertness, high hardness, high wear resistance, and low friction), which has made DLC a longstanding protective coating for surfaces of hard drive platters [179,180] and a leading candidate for solid lubrication of micro-devices [181]. In addition, DLC can be successfully applied to MEMS using a conformal, plasma-enhanced, chemical vapour deposition technique (CPI/CVD) [79,182]. This technique is also capable of depositing the DLC on underside structures of MEMS [79].

#### 5.3.2. Friction and Wear Behavior

DLC coatings are generally harder compared to other solid lubricants or even hard metals [183]; hardness values of DLC coatings have been reported up to 27 GPa measured with nanoindentation instruments [49,133]. Kuster et al. [133] performed a detailed study on the mechanical and microtribological properties of DLC coatings using the Hysitron nanoindentation instrument and a similar experimental procedure as described in the previous section. The main purpose of their study was to identify the fundamental wear and friction behavior of diamond-like carbon coatings using new experimental methods and analysis. The authors investigated DLC coatings on various materials currently used in hard drives and microdevices. More specifically, the DLC coatings on Si substrate experienced the least wear and smallest amount of plastic deformation compared to DLC on NiFe, NiFe, and SU 8 on NiFe. Similarly, the coefficient of friction for the DLC on Si sample was 0.08, which was lower compared to the other materials that were tested.

**Figure 14:**
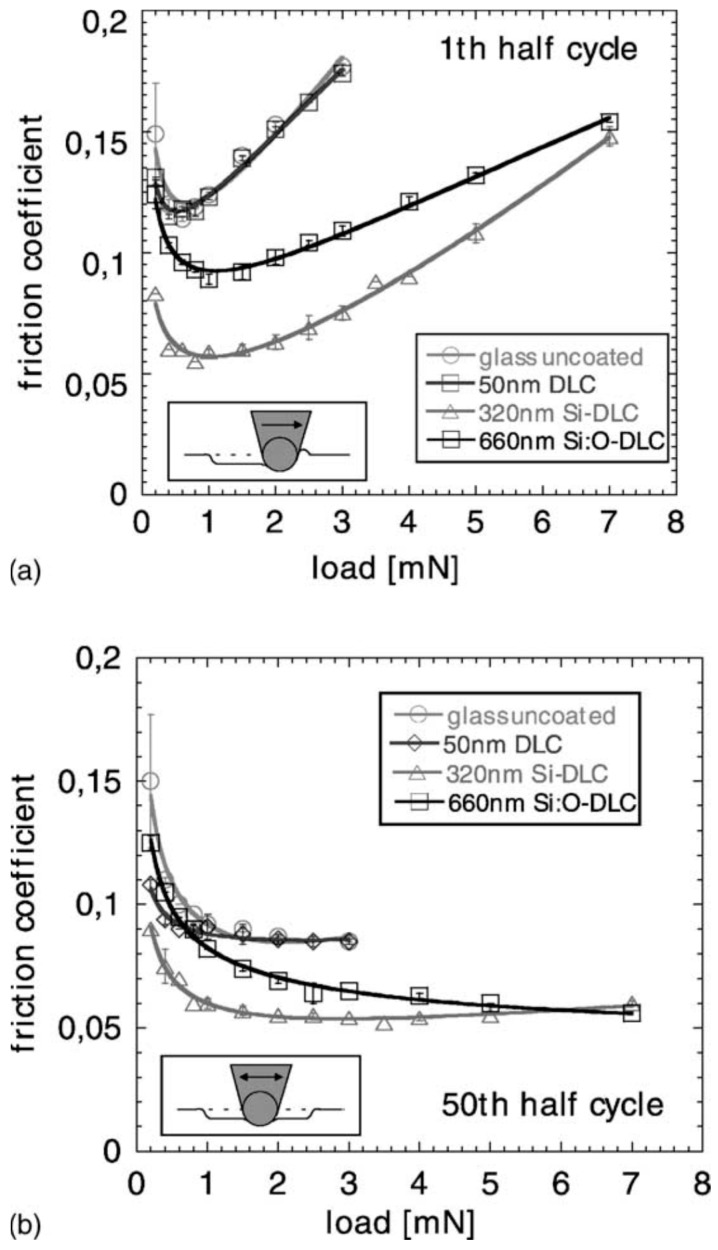
Coefficient of friction vs. load for diamond like carbon (DLC) coatings (**a**) 1st half cycle and (**b**) 50th half cycle. The data is fitted according to Equation (1). It is observed that for the higher cycle numbers the friction remains relatively constant for the higher load regimes, Figure 14b; For the first few cycles on the other hand, the friction increases with the higher load regime, which resulted in higher plowing exponent *m*, Figure 14a (Adapted from reference [127]).

Using the same nanoindentation instrument and experimental methods, Schiffmann et al. [127] explored the different contributions to the coefficient of friction, using Equation (1), on different types of DLC coatings. The authors showed that with the lower load regimes the coefficient of friction was dominated by the elastic component and after a critical load the plowing component increases. This was explained by the fact that with the lower normal loads the tip is mostly sliding on the surface and with the higher normal loads the tip is plowing through the coating. Using the same equation, Schiffmann was also able to show that the coefficient of friction is mainly dominated by the plowing component in the first few cycles of the test, whereas with the higher cycle number, the elastic component increases and eventually dominates, as shown in Figure 14. In terms of the coatings performance in this study, the Si doped DLC coating showed the lowest coefficient of friction (i.e., *µ* = 0.05–0.06) and lowest amount of wear (plastic deformation and removed material) compared to pure DLC, silicon-oxide doped DLC, and uncoated glass.

**Figure 15:**
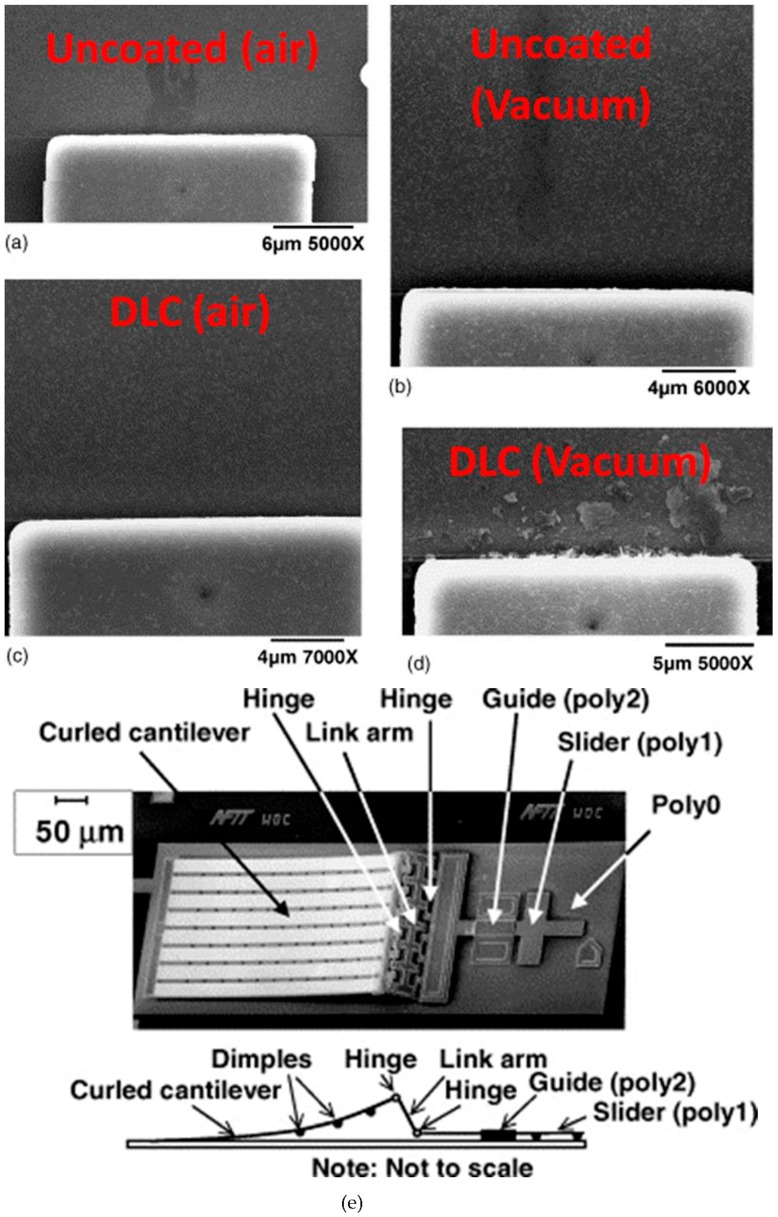
SEM of the slider of the electrostatic lateral output motor after the tests for (**a**) uncoated in air; (**b**) uncoated in vacuum; (**c**) DLC coated in air; (**d**) DLC coated in vacuum; and (**e**) an electrostatic lateral output motor as well as a cross sectional schematic figure of the microsystem (Adapted from reference [79]).

Smallwood et al. [79] deposited DLC coatings on actual MEMS devices and evaluated their performance and failure mechanisms. As a MEMS device, the authors used the electrostatic lateral output motor which is shown in Figure 15. This MEMS device provides many opportunities for characterizing the wear behaviour due to the numerous contact locations (e.g., hinge area and dimples that make the contact). The first wear analysis in this study was performed on the wear track and the slider, as shown with the SEM image in Figure 15, and the second analysis on the hinge region of the MEMS. Comparing the different conditions, slight wear tracks were observed for the uncoated MEMS device that was run in air, whereas the DLC coated system revealed nearly no signs of wear. Most debris particles for all conditions were observed in the hinge region, where the greatest number of contact is present and also where catastrophic failure was observed. In air, the DLC coated systems revealed a six times increase in performance over the uncoated systems, whereas in vacuum the DLC coated MEMS performed up to three hundred times better over the uncoated ones. Similarly to the study with the nanoindentation instrument by Kuster et al., the DLC coatings here showed excellent wear resistance and low friction values.

Not surprisingly, the high wear resistance of the DLC coating in this study was also consistent with earlier studies on microtribological behaviour for DLC [105,169,170,172]. Beerschwinger et al. [169] used a specimen-on-disk apparatus that is capable of simulating contacts and normal loads observed with microdevices to study the wear resistance of materials used in MEMS. The highest wear resistance was observed with DLC on DLC sliding (i.e., the wear rates for DLC on DLC sliding were three orders of magnitude lower compared to polysilicon on Si_3_N_4_).

#### 5.3.3. Understanding of the Tribology through 3rd Bodies

Beerschwinger et al. [169] identified two dominant wear mechanisms at the micro-scale; asperity fracture and asperity deformation. However, it has been frequently proposed that the improved tribological properties at the microscale of DLC coatings are attributed to a decrease in adhesive forces and therefore can be used as antiahesion coatings for MEMS/NEMS at various environmental and operating conditions. This was also confirmed with the study performed by Liu et al. [105], who showed that DLC significantly reduced the surface adhesion compared to Si(100) and is less sensitive to rest time, sliding velocity, and relative humidity.

#### 5.3.4. Solutions/Improvements

Although DLC coatings have a great potential for applications in MEMS due to their low adhesion and friction forces, there have been some setbacks. One of these issues is with the deposition processes, which are not compatible on certain microdevices. In addition, due to the small sizes and design constraints of MEMS, coatings applied to these systems are prefered to be relatively thin. However, the tribological properties of DLC coatings with a thickness below 300 nm can be influenced by the substrate materials and the topography. Bandorf et al. [166] showed that higher wear and lower friction is obtained with softer substrates, but suggested that in order to opimize the properties the whole system should be considered (i.e., chemistry of substrate and coating).

Keeping the thickness issues of these coatings in mind, researchers have recently focused on graphene as an alternative to DLC for applications in microsystems. Using a Basalt Must tribometer, Marchetto et al. [118] studied the microtribological behavior of epitaxial graphene grown by thermal deposition on SiC. The authors found that while the graphene layer is damaged during reciprocating sliding, the coefficient of friction is still lower compared to graphite and five times lower compared to hydrogen etched SiC.

### 5.4. MoS_2_ Based Coatings

#### 5.4.1. Technology Relevance

More recently, other materials besides DLC have also become of interest in microtribological applications. Researchers have demonstrated how the atomic-layer deposition technique can be used to successfully depositing chalcogenides onto microdevices [184,185] and therefore have raised the interest of studying the microtribological properties of solid lubricants such as WS_2_ and MoS_2_. Similarly to DLC, the behaviour of molybdenum disulphide based coatings at the micro-scale have been studied intensively due to their low friction values at the macro- length scale [40,41,42,43,186,187,188,189].

#### 5.4.2. Friction and Wear Behavior

Sahoo et al. [113] used lateral force microscopy (LFM) and the CSM nanotribometer to explore the frictional performance of molybdenum disulphide particles sprayed on smooth steel surfaces. Using these instrumentation, the authors investigate the microtribology of large (~2 µm) and small (~50 nm) particles with a wide range of contact pressures and normal loads. The friction coefficient was found to be lower with the 2 µm size particles compared to the 50 nm particles for all contact pressures and velocities. This was explained by the adhesion properties of the particles and their ability of the particles to stay in the contacts. In addition, from the micro-scale friction results in this study, it was observed that the coefficient of friction decreased with increasing the normal load and contact pressure.

**Figure 16:**
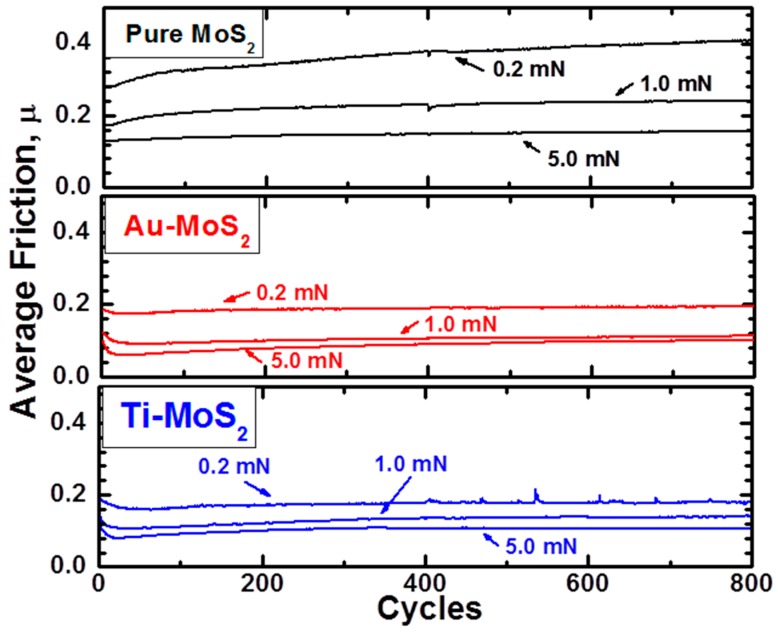
Average coefficient of friction vs. cycle number for a spherical diamond tip (R = 50 μm) sliding against Pure MoS_2_, Au-MoS_2_, and Ti-MoS_2_ using a normal load of 0.2 mN, 1.0 mN, and 5.0 mN (Contact Stress: 0.4–1.2 GPa). The coefficient of fricition decreases with the addition of Ti and Au to MoS_2_ for all normal loads tested. The lower friction values with the metal doped coatings is attributed to an increase in mechanical properties, a decrease in surface adhesion, and a decrease in surface adhesion (adapted from reference [21]).

Lower friction coefficient with higher normal loads has also been observed at the micro-scale with MoS_2_ based thin film coatings of thicknesses between 300 and 900 nm [21]. This microtribological behaviour was explored using a nanoindentation instrument on pure MoS_2_, Ti-MoS_2_, and Au-MoS_2_ coatings. Examples of the coefficient of friction vs. cycle number are shown in Figure 16 for sputtered MoS_2_, cosputtered Au-MoS_2_, and cosputtered Ti-MoS_2_ respectively. The friction results in these plots were obtained for a variation of normal loads (i.e., from 0.2 mN to 5.0 mN) using a spherical diamond tip with a 50 µm radius. A summary and analysis of the result in this study are shown in Table 4. The velocity accommodation parameter (i.e., interfacial shear strength), *S*_o_ was calculated using Equation (2) and the real contact area for the contact pressure *P*. The *S*_o_ value was found to decrease with the addition of metal content, which correlated with a decrease in wear volume and friction coefficient, Table 4. In terms of the relationship between the friction force and the normal force (i.e., *F* α *L^m^*) the pure MoS_2_ sample shows the closest value to 2/3, when compared to the two other coatings. It was concluded that the improved tribological performance with the metal doped MoS_2_ coatings was attributed to an increase in mechanical properties, a decrease in surface adhesion, and a decrease in surface adhesion.

#### 5.4.3. Understanding of the Tribology through 3rd Bodies

**Figure 17:**
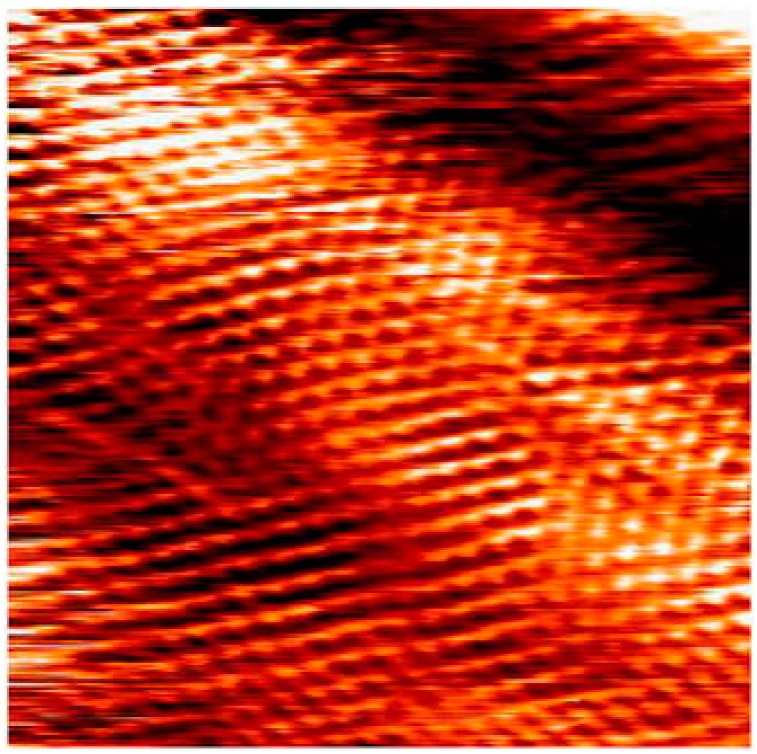
Topographical image of a MoS_2_ tribofilm on nanocomposite Au-MoS_2_film (7 nm × 7 nm). The image was produced using an atomic force microscope and shows the high -resolution crystal structure of the tribofilm. The lattice structure corresponds to that of a single crystal MoS_2_ with the basel planes parallel to the surface (adopted from reference [190]).

To obtain further insights on what is ‘really’ happening in the buried sliding interfaces of MoS_2_-based coatings at small length scales, Kim et al. [190] studied the lubrication mechanism of cosputtered Au-MoS_2_ coatings using a conductive atomic force microscopy (c-AFM). The authors were able to provide a visualization of the tribofilm on the worn surface of the Au-MoS_2_ nanocomposite film, as shown in Figure 17. The tribofilm consisted of crystalline MoS_2_ with the basal planes parallel to the sliding direction, which is consistent with literature on tribological properties of Pb-MoS_2_ and PbO-MoS_2_ coatings at the macroscopic scale. It was concluded in this study that the tribofilm formation at the nanoscale is responsible for the low friction and high wear resistance of MoS_2_ based coatings.

The evolution of the crystalline MoS_2_ tribofilm with respect to the friction behaviour, at the small length scale, was further explored using Raman spectroscopy [21,22,25]. Previous macrotribological studies [42] have shown Raman spectroscopy to be an effective analysis technique for identifying crystalline orientation of MoS_2_ based films. Thus, ex situ micro-Raman analysis on worn surfaces, which were created using a microtribological instrument, were performed metal-doped MoS_2_ coatings with varying metal content. For a variety of contact pressures, Raman peaks that are consistent with crystalline MoS_2_ were observed on the worn surfaces and were not seen on the as deposited amorphous coatings. This was an indication that a MoS_2_ tribofilm, similar to the one observed by Kim et al., was formed on the worn surface of MoS_2_-based coatings. These MoS_2_ tribofilms have thicknesses of a few nanometers and typically have their basal planes parallel to the sliding direction [40,43].

### 5.5. Gold Coatings

#### 5.5.1. Technology Relevance

Gold is a noble material and is known for excellent corrosion resistance, great electrical conductivity and thermal properties [49]. It is currently a widely used material in contact switches, where adequate electrical properties are crucial [3,191,192]. Dugger proposed [193] that possibly the largest market for microdevices in the near future is with microswitches (e.g., DC motor controls and RF micro-electromechanical systems). These devices offer essential advantages over conventional diode-type switches [193], including lower power loss, which can increase the battery life in certain systems (e.g., cell phones). Failure mechanisms in these microswithes devices typically include adhesion, melting, and increase in electrical resistivity due to coating (i.e., low-resistivity material) failure [22]. Therefore, there have been numerous studies on the mechanical and tribological properties of Au with the purpose to improve its reliability in microsystems.

#### 5.5.2. Friction and Wear Behavior

Such a study on the mechanical and tribological behaviour of gold and copper coatings for applications in RF MEMS switches was performed by Barriga et al. [23] using a nanotribometer (CSM Instruments). The microtribological tests were performed with varying the normal load between 1 and 20 mN at 33% and 84% relative humidity. The authors concluded that for Cu-Cu contacts the coefficient of friction at the microscale was affected by relative humidity (i.e., friction increased with increasing the relative humidity of 50%), whereas the friction coefficient for Au-Au contacts remained nearly unchanged with varying the humidity level between 33% and 84%. The increase in the friction coefficient for the Cu-Cu contact was explained due to capillary forces at the higher relative humidity level.

One successful lubrication strategy with the purpose to improve the mechanical and tribological properties of gold was developed by Lince [194,195], who cosputtered Au with a small amount of MoS_2_. In a macrotribological study, the authors reported that the best performance (i.e., lowest friction and highest endurance) for low stress regimes was observed with Au coatings containing between 10 and 25 mol % MoS_2_ [195]. In addition, the smallest increase in electrical resistivity compared to pure Au coatings, was seen by the 20 mol %MoS_2_-Au coatings. Therefore, this coating showed the most promise for applications in microswitches and was further investigated with instruments that simulate real MEMS contacts [22].

**Figure 18:**
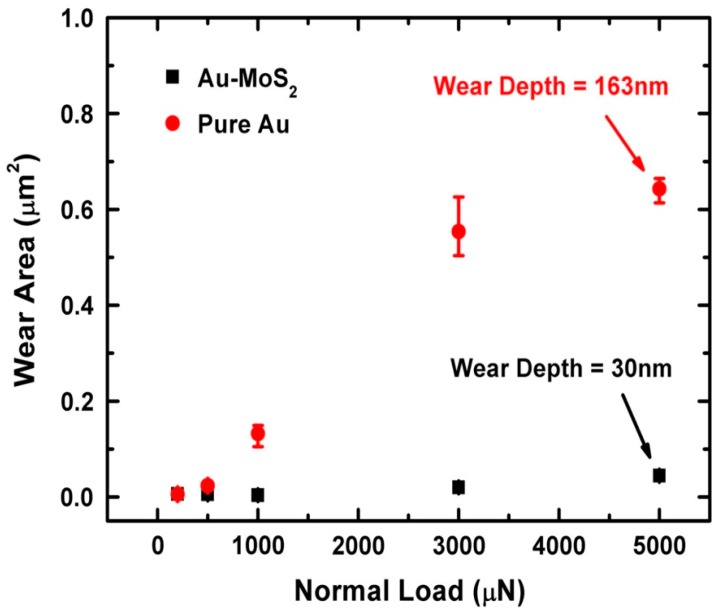
Wear area vs. normal load for microtribological experiments of a spherical diamond sliding against pure Au and Au-MoS_2_ coatings. The small addition of MoS_2_ (20 mol %) drastically decreases the wear area compared to pure MoS_2_ coatings. The higher wear resistance with the Au-MoS_2_ coating compared to the pure Au coating was attributed to a decrease in surface adhesion and differences in velocity accommodation modes (adopted from reference [22]).

In this study, MEMS contacts were simulated using a Hysitron nanoindenter with a 50 µm spherical diamond tip [22]. The mechanical and tribological performance of the Au-MoS_2_ coating were compared to pure Au coatings with varying contact sizes and pressures. The small addition of MoS_2_ to Au was found to increase the wear resistance significantly for all normal loads, as shown in Figure 18. The higher wear resistance with the Au-MoS_2_ coating compared to the pure Au coating was attributed to a decrease in surface adhesion and differences in velocity accommodation modes. This article concluded that the small additions of MoS_2_ to Au could be a helpful for microcomponent and microswitch applications with sliding interfaces.

Another approach to address reliability issues in microswitches was proposed with the addition of a MoS_2_ sacrificial layer on top of Au coatings [129]. The basic idea of this lubrication strategy was for the MoS_2_ capping layer to ‘fail’ early on in the operation stage of a microdevice, allowing for electrical conduction to occur, but leaving behind a very thin MoS_2_ tribofilm that would still protect the Au surface with minimal impact on electrical performance. In addition, this layer would promote the formation of a transfer film on the counterface which would further increase the wear resistance. These coatings were tested using three different microwear experiments (classified as 1D and 2D tests) and were compared to pure Au coatings. The results of this study showed that the bilayer coatings (i.e., Au with MoS_2_ capping layer) improved the tribological properties and therefore are also potential candidates for microtribological systems with sliding interfaces.

### 5.6. Microtribology of Polymers

#### 5.6.1. Industrial Relevance

The recent advances in BioMEMS/ NEMS has led to intensive research on exploring materials that can potentially replace silicon as the primary material choice. While the silicon micromachining processes are currently well developed, they are expensive, time consuming, and require special facilities [196,197]. In addition, silicon-based bioMEMS have revealed challenges with moving components and therefore limits the design opportunities. Polymeric materials, overcoming most of these challenges, have therefore received a great amount of attention for bioMEMS [196]. Besides being more costly beneficial, the machining and manufacturing process of polymeric materials is significantly easier compared to silicon. Moreover, polymers are ideal for bioMEMS due to their increased wear and corrosion resistance, compared to silicon. The wide variety of polymeric materials makes it also easier to select a material with the desired mechanical properties for a specific application.

Most literature on studying the friction and wear properties of polymeric material has used macro-scale tribological equipment [198] mostly because of the interest for biomedical joint applications. However, it is certainly possible that the wear behavior of polymers also varies with decreasing the contact size down to a few micrometers. Therefore, more studies have recently focused on investigating the tribological properties of polymers at the microscopic length scales in order to understand the scale effects [199] and their range of applications in bioMEMS.

#### 5.6.2. Friction and Wear

Tambe et al. [196] studied the scale dependence of poly(dimethylsiloxane) PDMS and poly(methylmethacrylate) PMMA on the adhesion and friction properties using a microtribometer and an AFM. The authors showed that the friction values were smaller with the polymers compared to the silicon. In addition, PDMS and PMMA showed no dependence of the holding time and relative humidity on the adhesive forces, while adhesion force of Si (100) was strongly dependent on the rest time and humidity levels. Therefore, the obtained results in this study suggested that these polymeric materials are ideal for applications in bioMEMS, where significant variations in environmental conditions and rest times are present.

Microscale abrasive wear for a wide range of polymeric materials was also studied by Shipway et al. (198) in order to understand the wear rate with small size abrasives (2–5 µm). The authors used a micro-scale abrasion tester to investigate the wear behavior of HDPE, PC, PETG, PMMA, PP, PS, and PVC. One of the conclusions of this study was that the wear rate has a strong dependence on the polymer type and its mechanical properties (e.g., hardness). It was pointed out that the polymers with small hardness values experienced a low wear coefficient value and the polymers with high strain to failure showed less indention induced wear.

While most of the attention on polymer brushes has been given in studying the nano- [200,201,202] or macro- scale [203,204,205] tribological properties, some studies have also been recently performed at microscopic length scales [199]. Using a silica probe particle with a 10 µm diameter (i.e., also modified with polymer brushes), concentrated polymer brushes (CPBs) revealed extremely low friction values (i.e., *µ* < 5 × 10^−4^) which were relatively independent of the normal load. Semi-dilute polymer brushes (SDPBs), on the other hand experienced low to high-frictional stages with an increase in normal load.

#### 5.6.3. Understanding of the Tribology through 3rd Bodies

Compared to macroscopic tribological behavior of CPBs, Kobayashi et al. [204] showed slightly higher friction values. However, Tsujii et al. [199] suggested that the sliding mechanism should be similar at both length scales. The authors found that the increase in friction values with the higher normal loads for SDPBs is attributed to the interpenetration of the brushes, while the low friction in CPBs is due to their large osmotic and elastic interactions (i.e., lack of intermixing).

In general, compared to silicon, the third body behavior of polymeric materials at the microscopic length scale is quite different [196]. The main contributions to the friction of silicon are meniscus bridges, mechanically deformed layer, debris particles, and the formation of Si(OH)_4_, whereas Tambe et al. [196] suggested that in the microtribology there is only localized melting and smooth sliding for PMMA and PDMS, respectively. Similarly, for microscale abrasive wear, Shipway et al. [198] saw that HDPE exhibited grooves in the parallel direction to the sliding and no evidence of rolling or ‘three -body damage’. However, other polymers in the same study (e.g., PMMA and PS) showed high wear due to damage induced by particle indentation through rolling.

### 5.7. Other Lubricants for MEMS

Besides the great potential in solid lubricants (e.g., DLC, MoS_2_) for applicaitons in microdevices, there is also a great interest in liquid crystals, fluid films, and other liguid/vapour phase lubricants to be used in MEMS [206]. Several studies have demonstrated how self-assembled monolayers (SAMs) can successfully be used on silicon based surfaces making the surface hydrophobic and preventing initial adhesion [207,208]. While hard coatings and solid lubricant encounter difficulties with various deposition techniques, SAMs do not have a significant effect on the packaging and encapsulation steps. Another great advantage of SAMs over hard coatings is that they do not face issues with the thermal coefficient of expansion upon exposure to various temperatures.

One limitation of self-assembled monolayers is their durability in complex microdevices that include sliding contacts and high contact stresses. Under these conditions, they wear off relatively quickly exposing the surface underneath (e.g., silicon oxide) and resulting in unacceptable adhesion and wear behavior. Using a MEMS switch simulator Patton et al. [209] studied the physical and chemical processes that occur in Au dc switch contacts coated with SAM. The authors identified different failure mechanisms when using the SAM coating and provided a good understanding of the direction for the development of reliable MEMS lubricants.

**Figure 19:**
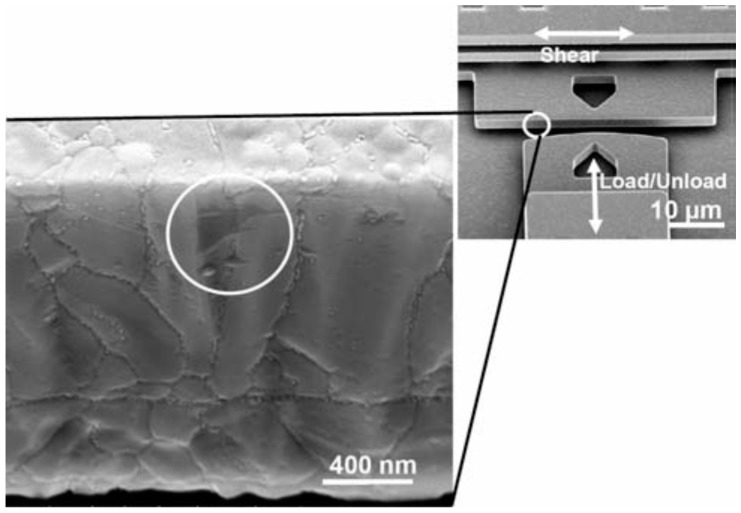
Ex situ SEM analysis of the MEMS sidewall surface after 1.02 × 10^8^ cycles in 1-pentanol environment. Even after such a long duration, no debris particles are observed. (Adapted from reference [210]).

Asay et al. [210] studied vapor-phase lubrication at the microscopic length scale in order to overcome the challenges observed with SAMs under long sliding periods. Following up on previous studies [211] that focused on the atomic scale sliding behavior (i.e., with AFM) of alcohol vapors, this work was performed using a sidewall MEMS device in order to note the performance of this lubrication strategy in microsystems. The authors found that 1-pentanol vapor significantly increased the endurance life of MEMS devices by maintaining its lubrication properties for long cycle numbers. This successful lubrication behavior was attributed to the ability of the alcohol to form and maintain a lubricating layer on the silicon surface [210]. Figure 19 shows an ex situ SEM analysis of the MEMS sidewall surface which ran for over 108 cycles. Even after such a long duration, no debris particles were observed. The authors also noted a ‘fluid-like’ deposit near the tall asperities [210].

In addition to studying vapor-phase lubricants as an alternative to SAMs, Patton et al. [209] and Voevodin et al. [212] investigated nanoparticle liquids (NPL) and compared theirmicrotribological performance to SAM of diphenyl disulfide. In this study, the authors focused on the lubrication and failure mechanisms of NPL-coated Au MEMS dc switch contacts. Compared to SAM- based switch lubricants, the NPL’s showed excellent durability and electrical properties indicating their potential candidacy for MEMS switch lubrication.

## 6. Comparison between Micro- and Macrotribology

In terms of solid lubrication, for macrotribology the contact area is significantly larger relative to the coating thickness, whereas in most microtribological studies the contact area is similar or larger to the thickness of the coating. Perhaps the most visual differences between the two scales are the normal loads and the contact areas. In macrotribological experiments (e.g., ball-on-disk), usually heavy loads are used (can range anywhere between 10^−3^ and 10^2^ mN), which typically result in large contact areas (1). For microscopic experiments (e.g., AFMs, MEMS tribometers, Microtribometers, etc.), on the other hand, the normal loads are much lighter resulting in Hertzian contact radii between 0.003 and 4 µm. Because of the significant variations in normal loads and contact areas between macro- and microtribology, the properties that dominate the tribological performances are different; at the macroscale the tribological performance are dependent on the bulk material properties, whereas for microtribology the surface properties dominate the friction and wear behaviour. These differences between macro- and microtribology can certainly result in variations of the friction coefficient, wear rate, interfacial shear strength, surface adhesion, velocity accommodation modes, etc.

Compared to macrotribological studies, the friction values for DLC coatings tested at the microscale seem relatively high; macro-friction of such coatings is in the range of 0.001 to 0.005 [49,213]. The higher friction values at the micro-scale compared to macro-scale could possibly be explained by plowing effects due to tip roughness and tip radius (i.e., R = 2.6 µm), surface adhesion effects, absence of transfer films, differences in environmental effects, and differences in localized contact pressures.

It is generally true for macro-scale sliding on DLC coatings that transfer- and tribo- films are formed after a given number of cycles at which state minimal wear and nearly Hertzian behaviour is observed (i.e., *µ* = *S*_o_/*P*_H_ + α, where *P*_H_ is the Hertzian contact stress). The transfer film and the formation of a graphitic layer on top of the hard coating contribute to the low friction coefficient of DLC [49]. In situ studies revealed that fluctuations in friction regimes are attributed to unstable transfer film [147]. In addition, for macrotribological studies, the main velocity accommodation mode is observed to be interfacial sliding between the transfer film and the counterface when friction is low (*µ* ~ 0.05). When the friction coefficient increases, however, a second velocity accommodation mode appears; transfer film shearing. The additional velocity accommodation mode with the increase in the friction values also correlates with an increase in the interfacial shear strength *S*_o_ value according to Equation (2).

At the microscale, on the other hand, Schiffmann et al. observed an additional velocity accommodation mode, which was plowing. This VAM was dominating the coefficient of friction more so throughout the first cycle and was less dominant during steady state sliding. The plowing exponent *m* from Equation (1) was found to be in the range of 0.7–1.2 and 0.13–0.68 for the first few cycles and the steady state sliding (i.e., 50th pass) respectively.

A more direct comparison between macro- and micro- scale tribology of DLC coatings was performed by Xie et al. [178]. The authors used a friction force microscope (FFM) with different probes and compared the friction performance to results obtained with a ball-on-disk tribometer. The FFM results showed that the coefficient of friction is influenced mainly by surface adhesion and topography at the microscale. In addition, the build up of debris particles seem to also have a significant effect on the friction behaviour at the micro scale.

Other influencing factors on the tribological behaviour at the microscale were investigated also with respect to the contact area and contact pressure [166]. For thin DLC coatings (i.e., below 300 nm), the friction coefficient decreases with decreasing the contact radius. Additionally, with such thin coatings, the properties of the substrate can have a significant effect on the friction and wear behaviour for micro contacts; the friction decreases and the wear resistance increases with decreasing the young’s modulus of the substrate.

These influencing factors on the microtribological properties are not necessarily true for macro-scale contacts. For example, a study comparing substrate effects on the macrotribological properties of DLC coatings also showed that variations in the wear resistance arise from differences in mechanical properties of the substrate [214]. However, unlike with microtribology, the highest wear resistance in this study was obtained with the substrate having the highest elastic modulus (i.e., M42 tool steel).

**Figure 20:**
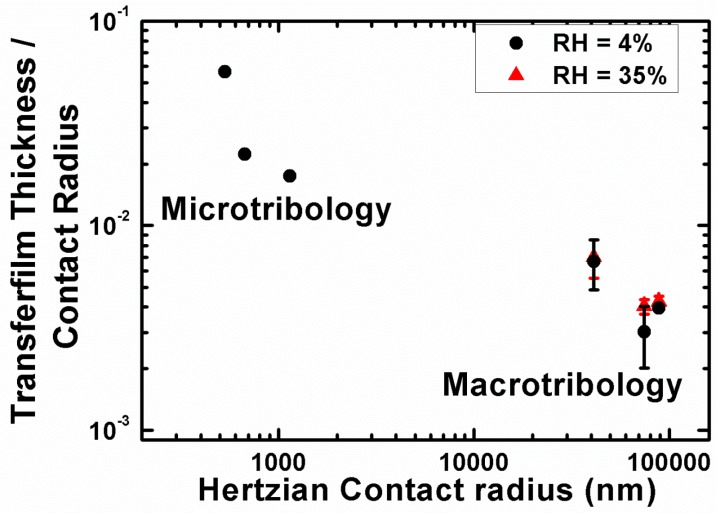
Transfer film thicknesses of micro- and macrotribological experiements normalized to the Hertzian contact radius versus the Hertzian contact radius. The transferfilm thickness for the tests at microscale were measured ex situ, while the thicknesses for the macrotribology were obtained using the in situ methodology presented in Figure 13. The normalized transfer film thickness for various contact pressures was lower for macrotribology compared to dry sliding with microtribology. For microtribology in humid environments, the transferfilmtransfer film was undetectable (Adapted from reference [25]).

A similar correlation is observed with the friction and transfer film behaviour between micro- and macro- scale tribology of MoS_2_ [25]. The friction coefficient at the micro-scale (i.e., contact radius is between 0.39 and 1.15 µm) has been observed to be higher compared to macro-scale friction (i.e., contact radius is between 30 and 90 µm), which correlates with the differences in the normalized transfer film thicknesses between the two scales. Figure 20 shows transfer film thicknesses normalized to the Hertzian contact radius versus the Hertzian contact radius. The normalized transfer film thickness for various contact pressures was lower for macrotribology compared to dry sliding with microtribology. For microtribology in humid environments, the transfer film was undetectable and therefore not thick and consistent enough to cover all asperities on the slider. This absence of transfer film with humid sliding changes the velocity accommodation mode to plowing. The plowing event with microtribology at higher humidity levels was also confirmed by fitting the data with Equation (1). Similarly to the analysis of Schiffmann et al. on DLC, the exponent *m* for MoS_2_ based coatings was in the range between 0.3 and 1.0 for high humidity tests. However, for lower humidity tests, the values of the exponent *m* were significantly lower and *L^m^* becomes nearly one indicating mainly elastic sliding.

The direct comparison between macro- and micro-tribology using in situ and ex situ analysis techniques led to the identification of three different stages for solid lubrication with respect to contact areas, tip shapes, and environmental conditions, Table 5. The first stage is the solid lubrication stage, which is found at macroscale for MoS_2_ based coatings under ideal conditions (e.g., spherical tip shape, dry air). Throughout this stage, a stable transfer film is formed and the friction behaviour is nearly Hertzian. The second stage was observed for microtribological experiments under dry conditions. In this stage stable transfer films are still observed, however the friction behaviour is non-Hertzian with some microscopic plowing events. The final stage of the sliding behaviour was observed with microtribology sliding in humid environments. During this stage, there was no stable transfer film observed and the main velocity accommodation mode is plowing (values of *m* in Equation (1) are between 0.3 and 1.0).

Similar contact scaling effects to the ones of MoS_2_ have also been observed on the fretting wear behaviour of AISI 52100 [215]. Fretting occurs due to small oscillatory motion of two contacting surfaces and may result in fatigue surface damages or surface wear caused by debris generation. Thus, fretting wear is also dependent on third body formation and/or chemical changes on the sliding interface. While keeping a relatively Hertzian contact pressure at 1.1 GPa, Marhej et al. [215] investigated the contact size behavior in fretting wear using different sphere radii and normal forces. The authors found that while the friction and wear is less affected by the sliding amplitude, the contact size has a significant impact on the fretting wear response of AISI 52100 against AISI 52100; the coefficient of friction and wear rates decrease with increasing the contact size. This behavior with respect to the contact size was explained by differences in the rheological properties of third body. Debris particles are not as easily ejected with larger contact sizes and the third body is thicker and more compliant compared to smaller contact sizes, which results in a better accommodation of the shear and thus lower friction.

## 7. Conclusions and Future Outlook

This review paper has addressed and outlined fundamental questions regarding the tribological properties at the microscopic scale. There exists numerous opportunities that can broaden our understanding of the sliding processes that control the friction and wear properties at the microscopic length scale. By combining our finding of the interfacial phenomena at the macroscopic length scales (e.g., using in situ tribometry) with new methodologies at the micro-lenght scale, one can better understand and test the sliding behaviours using small contacts. As a future outlook, conducting in situ nano-/microtribological experiments using transmission electron microscopy or scanning electron microscopy is now possible and future research will provide a better understanding on the interfacial phenomena and velocity accommodation modes of microcontacts.

## Figures and Tables

**Table 1:** Summary of friction laws, adapted from reference [47].

Friction Laws	*F*_f_ vs. Area	*F*_f_ vs. *L*	Notes
Macroscale Theories			
Amontons‘ law	Independent of *A*_macro_	$F_{f}=\bar{\mu }\cdot L$	Law first discovered by Leonardo da Vinci
Bowden and Tabor	$F_{f}=\bar{\tau }\cdot \sum A_{asp}$	$F_{f}=\bar{\mu }\cdot L$	Law results from contact roughness
Single-Asperity Theories			
Non-adhesive (based on Hertz model)	$F_{f}=\tau \cdot A_{asp}$	$F_{f}\propto L^{2/3}$	Linear dependence of *F*_f_ on *A*_asp_ is generally believed to be true for microscale contacts, but has been questioned for nanoscale contacts
Adhesive (for example, Maugis–Dugdale)	$F_{f}=\tau \cdot A_{asp}$	Sublinear	–

**Table 2:** Common failure mechanisms of Microsystems (adapted from [54]). Failure modes that have either a primary or secondary relationship with tribological interactions are underlined.

Category of Failure	Types of Failure
Fracture	Overload Fracture Fatigue Fracture
Creep	Applied Stress Intrinsic Stress Thermal Stress
Stiction	Solid Bridging Capillarity force van der Waals force Electrostatic force Asperity deformation force Micromachine critical stiffness
Electromigration	–
Wear	Adhesive Abrasive Corrosive
Degradation of Dielectrics	Leakage Charging Breakdown
Delamination, Contamination, Pitting of Contacting Surfaces, and Electrostatic Discharge	–

**Table 3:** Micro-electromechanical systems (MEMS) tribometers and their capabilities.

Type	Capability	Activation	Interfaces
(First) MEMS tribometer -1990 [62]	-Measurement of friction using restoring force of a displaced spring	Electrostatic attraction	-polySi * on Si_3_N_4_ -polySi on polySi
The Nanotractor [68]	-bi-directional motion -100 µm displacement range -high forces/velocities -study of friction and wear	‘Inchworm foot’	-monolayer coatings
MEMS electrostatic lateral output motor [69]	-wear behavior information at many contact locations -varying environmental conditions	Voltage applied to curled cantilever provides lateral motion	-bound/mobile hydrocarbon-based lubricants -Ionic-liquid lubricants -OTS self-assembled monolayer(SAM) coatings -DLC
Sidewall tribometer [70]	-first microdevice to measure kinetic friction at realistic MEMS contacts/velocities -various environmental conditions	Two electro-static comb-drive actuators	-monolayer coatings -thin hard coatings
Leiden MEMS tribometer [71]	-variety of tip shapes to be used -large range of normal forces -studying complex nanotribological effects -friction loops output	-Applied voltage to normal/lateral comb drive	-silicon oxide surfaces

* polySi = polycrystalline silicon.

**Table 4:** Microtribological properties summary for the experiments shown in Figure 16 for a diamond spherical tip sliding against pure MoS_2_ and metal doped MoS_2_. The addition of Ti and Au to MoS_2_ drastically increases the wear resistance and the interfacial shear strength, which is calculated using Equation (2).

Solid Lubricant	Wear Volume (µm^3^) (L: 3.0/5.0 mN)	*S*_o_ (MPa)	Friction (μ) (L: 3.0/5.0 mN)	Friction/Load Relationship
MoS_2_	1.1/1.9	39 (±6)	0.15/0.14	*F* ∝ *L*^0.78^
Au-MoS_2_	0.3/0.5	23.2 (±0.4)	0.10/0.10	*F* ∝ *L*^0.79^
Ti-MoS_2_	0.1/0.2	15 (±3)	0.11/0.10	*F* ∝ *L*^0.84^

**Table 5:** Stages of the sliding behaviour of MoS_2_ based lubricants with respect to contact areas, tip shapes, and environmental conditions. These stages were identified based on micro- and macro-scale tribological experiments. (Adapted from reference [25]).

-	Stage I: Solid Lubrication	Stage II: Micro-Plowing	Stage III: Plowing
Limiting Friction (α)	~small	~big	~L^m^
Friction behavior	Hertzian	non-Hertzian	non-Hertzian
General sliding behavior	solid lubricant	solid lubricant	not solid lubricant
VAM	interfacial sliding and/or interfilm shearing	interfacial sliding + micro-plowing	Interfacial sliding + plowing
Wear mechanism	adhesion	micro-plowing + adhesion	plowing
Tribofilm Formation ^	Yes	Yes	No
Transfer Film Present	Yes	Yes	No

^ Evidence of increased crystallinity of MoS_2_ from Raman spectroscopy.
